# Separation of Benzene and Cyclohexane Using Eutectic Solvents with Aromatic Structure

**DOI:** 10.3390/molecules27134041

**Published:** 2022-06-23

**Authors:** Mohamed K. Hadj-Kali, M. Zulhaziman M. Salleh, Irfan Wazeer, Ahmad Alhadid, Sarwono Mulyono

**Affiliations:** 1 Chemical Engineering Department, King Saud University, Riyadh 11421, Saudi Arabia; iwazeer@ksu.edu.sa (I.W.); sarmulyoprayitno@ksu.edu.sa (S.M.); 2 Department of Chemical and Process Engineering, Faculty of Engineering and Built Environment, Universiti Kebangsaan Malaysia, Bangi 43600, Malaysia; 3 Biothermodynamics, TUM School of Life Sciences, Technical University of Munich, Maximus-von-Imhof-Forum 2, 85354 Freising, Germany; ahmad.alhadid@tum.de

**Keywords:** ionic liquids, eutectic solvents, benzene, cyclohexane, LLE, COSMO-RS

## Abstract

The separation of benzene and cyclohexane is a challenging process in the petrochemical industry, mainly because of their close boiling points. Extractive separation of the benzene-cyclohexane mixture has been shown to be feasible, but it is important to find solvents with good extractive performance. In this work, 23 eutectic solvents (ESs) containing aromatic components were screened using the predictive COSMO-RS and their respective performance was compared with other solvents. The screening results were validated with experimental work in which the liquid–liquid equilibria of the three preselected ESs were studied with benzene and cyclohexane at 298.5 K and 101.325 kPa, with benzene concentrations in the feed ranging from 10 to 60 wt%. The performance of the ESs studied was compared with organic solvents, ionic liquids, and other ESs reported in the literature. This work demonstrates the potential for improved extractive separation of the benzene-cyclohexane mixture by using ESs with aromatic moieties.

## 1. Introduction

The separation of aromatic-aliphatic hydrocarbon mixtures is a challenging process in downstream fuel processing. An ordinary distillation method is economically unviable because the two compounds often have boiling points close to each other, i.e., 80.1 °C for benzene and 80.74 °C for cyclohexane. Furthermore, the formation of azeotropes in some combinations leads to additional difficulties in process efficiency. This suggests the use of more advanced separation techniques, which are classified into three types [1,2]: liquid extraction (for 20–65% aromatics content), extractive distillation (for 65–90% aromatics content), and azeotropic distillation (for more than 90% aromatics content). The most commonly used solvents in these processes are conventional organic compounds, such as sulfolane, polyethylene glycols, tetraethylene glycol, di-methyl sulfoxide, N-methylpyrrolidone, and N-formylmorpholidone [2]. Despite their industrial use, the use of these solvents suffers from high energy consumption, high capital and operating costs, and process complexity [3]. Moreover, there is no feasible process for the separation of aromatics content below 20%.

Liquid–liquid extraction is an interesting process because it can be operated at ambient conditions. To achieve efficient extraction, the choice of solvent is a crucial step, as it should be chemically stable, non-corrosive, inexpensive and easy to obtain. Interestingly, ionic liquids (ILs) have been extensively researched for their potential use as alternative solvents in many applications. In addition to their chemical and thermal stability, ILs also possess particular advantages over conventional industrial solvents, especially due to their negligible vapor pressure, wide range, and tunable properties. Moreover, ILs have also shown promising results in the separation of aromatics and aliphatics [4,5,6,7].

The feasibility of IL as extraction solvents was usually evaluated based on the benzene distribution ratio and selectivity. Several ILs were reported to show good selectivity, such as 1-ethyl-3-methylimidazolium tetrafluoroborate [C_2_Mim][BF_4_], 1-alkyl-3-methylimidazolium hexafluorophosphate [C_4_mim][PF_6_], and 1-butylpyridinium tetrafluoroborate [Bpyr][BF_4_] [8,9]. [C_4_mim][PF_6_] remarkably showed extremely high solvent selectivity at low composition (<15%) of benzene in the raffinate phase, suggesting that it is suitable for the deep separation of benzene and cyclohexane [9]. The choice of ILs is not limited to selectivity. For example, [C_2_py][EtSO_4_] was selected due to its ease of preparation, market availability, and satisfactory results in the separation of benzene from its mixture with other aliphatic compounds [10,11]. Although ILs have higher extraction performance than organic solvents, their use is rather limited mainly due to their toxicity and high cost [12].

Eutectic solvents (ESs) have been identified as a promising alternative for many separation applications [13]. ESs have been mainly investigated in the fields of electrodeposition of metals [14], nanotechnology [15], and extraction and separation processes [16,17]. Recently, however, they have also received attention in the fields of solar energy [18,19], photosynthesis [20], and electrochemical sensing [21]. Some examples of the use of ESs in various applications are listed in Table S1. ESs are widely recognized as a new class of IL analogs because they share many features and properties with ILs, especially that they are liquid at ambient temperature [22,23]. However, ESs can be prepared by simple mixing of inexpensive and natural substances. ESs are eutectic mixtures containing a salt and a hydrogen bond donor, forming a mixture with a much lower melting temperature than the raw materials [24,25].

Although the advantages of ESs are recognized, there is little information on their potential use in the separation of benzene and cyclohexane. In our previous work [26], five ESs were preselected using the Conductor-like Screening Model for Realistic Solvation (COSMO-RS), namely tetrabutylammonium bromide:sulfolane (TBABr:sulf (1:7)), methyl triphenylphosphonium bromide:triethylene glycol (MTPPBr: TEG (1:4)), tetrabutylammonium bromide:triethylene glycol (TBABr: TEG (1:4)), choline chloride:triethylene glycol (ChCl: TEG (1:4)), and methyltriphenylphosphonium bromide:propanediol (MTPPBr: PD (1:4)). The ESs were found to be useful extracting solvents in the separation of benzene-cyclohexane mixtures by liquid–liquid extraction. Although the benzene distribution ratio was low, efficient extraction can be achieved by a multistage process [26]. In another study [27], two ESs, namely tetrabutylammonium bromide:sulfolane, TBABr:sulf (1:5), and trimethylamine hydrochloride:ethylene glycol, TMAHCl: EG (1:5) were selected to separate benzene and cy-clohexane mixtures. Similar to our results, the study showed that TBABr:sulf (1:5) is a promising solvent for the extractive separation of the benzene-cyclohexane mixture.

In liquid–liquid extraction, it is ideal to use an extraction solvent that achieves high values in both selectivity and distribution ratio. However, numerous reports have found that in most cases, the distribution ratio of an extraction solvent has an inverse relationship with selectivity. This relationship presents a clear challenge in finding a solvent with high values for both selectivity and distribution ratio. Therefore, the development and modification of the solvent used is an important task. In summary, three attempts have been made to obtain ILs with high selectivity and high distribution ratio [28]: (1) searching for new and unusual ILs, (2) mixing with green co-solvents, or (3) mixing ILs. Solvent mixing has attracted particular attention in the extractive separation of aromatic and aliphatic compounds and has been discovered as a new efficient and versatile technique to optimize extraction performance [29,30,31,32].

COSMO-RS has been widely used in many research computational aproaches due to its fast and reliable prediction capability. COSMO-RS combines statistical thermodynamics and quantum chemical calculations to predict the thermodynamic properties of solvents without using experimental data. Considering this advantage, many screening processes have been performed to help research groups find the best solvents for various applications, such as desulfurization and denitrification. The mathematical derivations for the interaction energies, chemical potential, and activity coefficient derived by the developers of COSMO-RS can be found elsewhere [33,34], and we have summarized the equations involved in our previous work [35,36,37]. COSMO-RS has been used for various thermodynamic predictions and validations in critical processes such as denitrification [38], desulfurization [39,40], and separation of aromatic and aliphatic mixtures [41,42,43]. In this work, the extraction performance of ESs with aromatic structure for the separation of benzene and cyclohexane mixtures was investigated using COSMO-RS screening and validation by experimental liquid–liquid extraction.

## 2. Results and Discussion

### 2.1. COSMO-RS Screening Results

**Figure 1:**
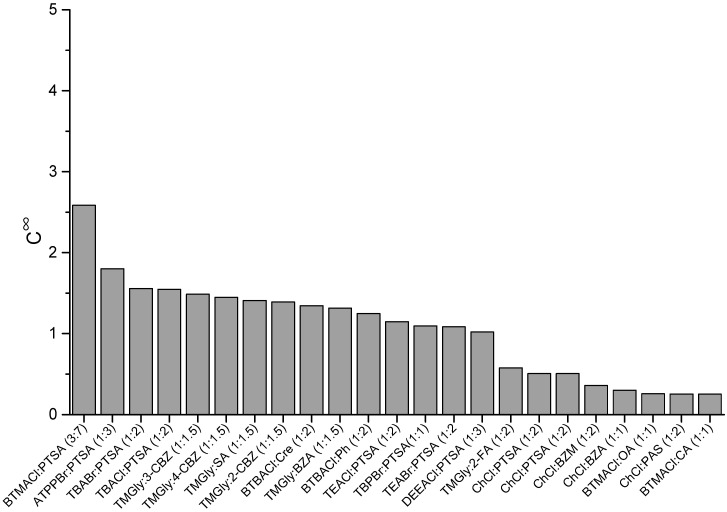
Capacity of ESs at infinite dilution.

**Figure 2:**
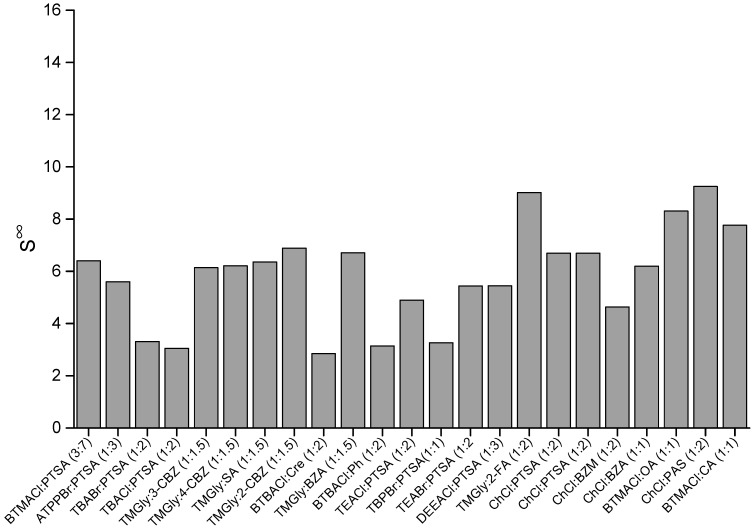
Selectivity of ESs at infinite dilution.

**Figure 3:**
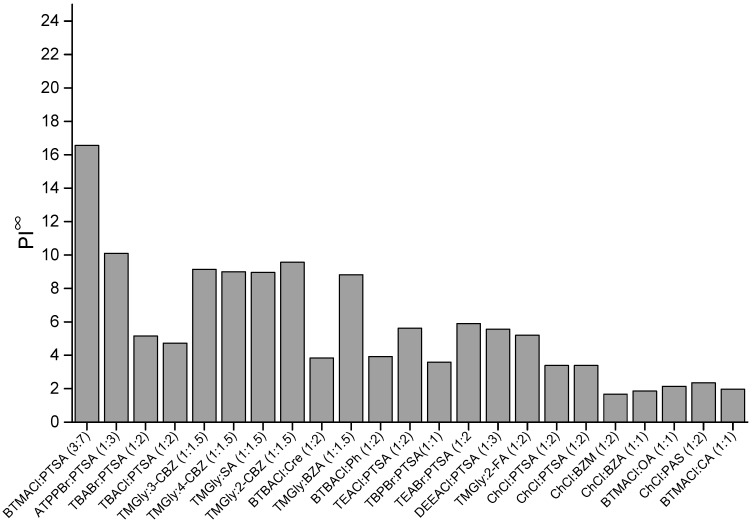
Performance index of ESs at infinite dilution.

In this study, COSMO-RS was used to screen the appropriate ESs by calculating their capacity (C∞), selectivity (S∞), and performance index (PI∞) at infinite dilution. The results of the ES screening are shown in Figure 1, Figure 2 and Figure 3. In these Figures, the studied ESs were sorted in descending order by the value of capacity at infinite dilution (C∞), since solvent capacity has been reported to have a much greater impact on production cost than selectivity. As seen in Figure 1, two ESs with different hydrogen bonding acceptors showed high C∞ values, namely BTMACl:PTSA (3:7) and ATPPBr:PTSA (1:3). In addition, a general trend was also observed for some ESs. TMGly generally showed a higher C∞ value compared to ChCl. The capacity of ESs generally increases with longer cation alkyl chain, i.e., TBABr:PTSA (1:2) > TEABr:PTSA (1:2) and TBACl:PTSA (1:2) > TEACl:PTSA (1:2).

As seen in Figure 2, the five ESs with the highest selectivity are in the order, ChCl: PAS (1:2) > TMGly:2-FA (1:2) > BTMACl:OA (1:1) > BTMACl: CA (1:1) > TMGly:2-CBZ (1:1.5). An inverse trend was generally observed when evaluating the capacity and selectivity of the same ESs. The capacity of an ES reflects its ability to extract other components regardless of their specific structure. On the other hand, selectivity evaluates the efficiency of the solvent in extracting the specific solute (benzene in this work). The predicted selectivity of the ESs in this work is generally higher than that of the ESs screened in our previous work [26], where the average selectivity increment is around 10.5%. This indicates the significant influence of the aromatic group on the selectivity. From this result, it can be concluded that the presence of an aromatic ring in the ES structure could enhance the selective interaction between ES and benzene through π-π stacking.

The performance index combines both selectivity and capacity by simple multiplication. Thus, the PI∞ is a mathematical operation that evaluates overall extraction performance by considering the inverse relationship between C∞ and S∞. As seen in Figure 3, five ESs with the highest PI∞ were in the order of BTMACl:PTSA (3:7) > ATPPBr:PTSA (1:3) > TMGly:2-CBZ (1:1.5) > TMGly:3-CBZ (1:1.5) > TMGly:4-CBZ (1:1.5). In liquid–liquid extraction, capacity indicates the amount of solvent required, while selectivity evaluates extraction efficiency. An ideal extraction solvent for efficient extraction would have high capacity and selectivity. However, due to inverse proportionality, it is difficult to obtain ESs with high values for both capacity and selectivity. Therefore, there is a conflict with one of these properties in the solvent screening process. Based on the results from COSMO-RS, BTMACl:PTSA (3:7) is expected to be the best extraction solvent because it has the highest value for both C∞ and PI∞. 

### 2.2. Experimental Selectivity and Distribution Ratio

In this work, experimental LLE was performed for the ESs obtained by mixing BTBACl with phenol or cresol in a molar ratio of 1:2 and for another ES, formed by mixing TBPB with PTSA in a molar ratio of 1:1. The LLE data at 298.15 K and 101.325 kPa were used to investigate the efficiency of benzene extraction from a benzene-cyclohexane mixture using the proposed aromatic-based ESs as extractants. The hydrocarbon-rich phase was analyzed by ^1^H NMR spectroscopy, and no ES constituents were detected. Accordingly, the two-phase system was assumed as a pseudo-ternary system, where the ES ratio remains constant. In this context, the efficiency of the extracting solvents was evaluated based on two important parameters: selectivity (S) and distribution ratio (D). Equations (1)–(3) were used to calculate the selectivity and the distribution ratio [26,44]:

(1) DBen=xBenBxBenT

(2) DCh=xChBxChT

(3) S=DBenDCh=xBenBxBenT×xChTxChB

where x is the concentration in mole fraction, Ben stands for benzene, and Ch for cyclohexane. Superscripts B and T refer to the bottom and top phases, respectively. 

**Figure 4:**
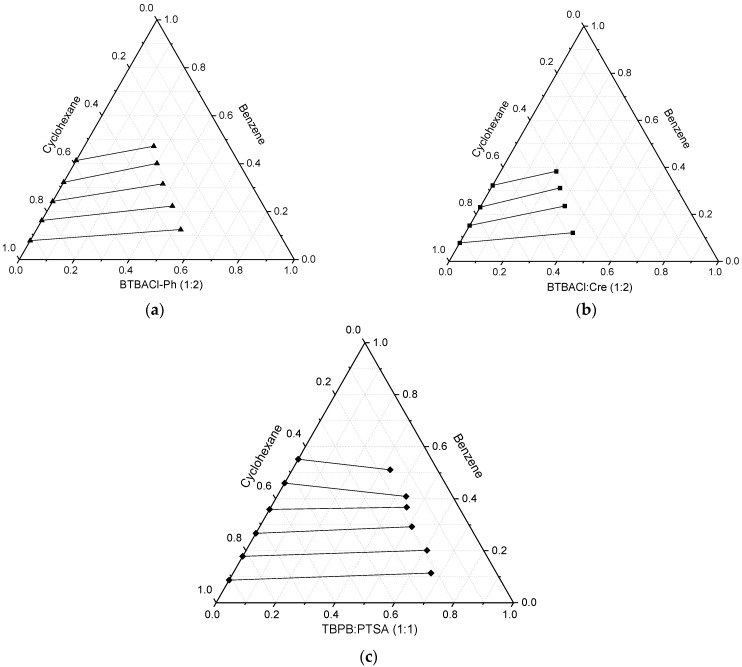
Ternary liquid–liquid equilibria diagrams for (**a**) BTBACl:Ph (1:2) + benzene + cyclohexane; (**b**) BTBACl:Cre (1:2) + benzene + cyclohexane and (**c**) TBPB:PTSA (1:1) + benzene + cyclohexane.

The LLE data along with the distribution ratio and selectivity of the three systems [benzene + cyclohexane + BTBACl + Ph (1:2, HBA:HBD molar ratio)], [benzene + cyclohexane + BTBACl + Cre (1:2, HBA:HBD molar ratio)], and [benzene + cyclohexane + TBPB + PTSA (1:1, HBA:HBD molar artio)] are shown in Table 1. *x*_HBA_ and *x*_HBD_ represent the molar composition of hydrogen bond acceptor and hydrogen bond donor, respectively. The experimental results of the three systems studied were plotted in ternary diagrams by treating the ESs as a single component in Figure 4 to graphically illustrate the phase equilibria. it can be observed that one pair of the components of all ternary systems exhibits partial miscibility with each other ([BTBACl:Cre or BTBACl:Ph + benzene or cyclohexane]), while the other pair of components exhibits complete miscibility ([benzene + cyclohexane]), i.e., in ternary systems consisting of BTBACl:Cre or BTBACl:Ph + benzene + cyclohexane, the binary compounds BTBACl:Cre or BTBACl:Ph + benzene and BTBACl:Cre or BTBACl:Ph + cyclohexane present partial miscibility, while the binary compound benzene + cyclohexane presents complete miscibility. It is also worth noting that for the first two ESs, i.e., BTBACl:Ph (1:2) and BTBACl:Cre (1:2), high concentrations of cyclohexane were consistently observed in the extract phase. This could be due to the benzyl group of the hydrogen bond acceptor interacting with cyclohexane such as the interactions in the benzene-cyclohexane mixture. The cross-solubility of cyclohexane in the ES phase would not cause much difficulty in the post-extraction system, since the ES can be recycled by heating off the cyclohexane due to the ES negligible vapor pressure.

**Figure 5:**
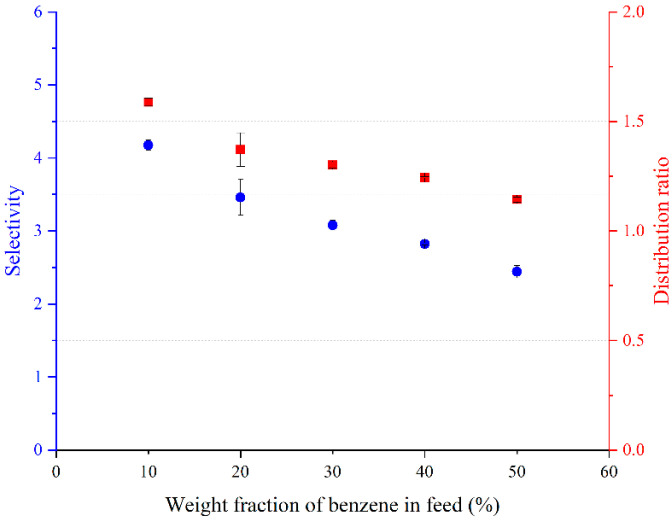
Variation of the distribution ratio and selectivity with benzene weight fraction in the feed for [Benzene (1) + Cyclohexane (2) + BTBACl:Ph (1:2) (3)].

**Figure 6:**
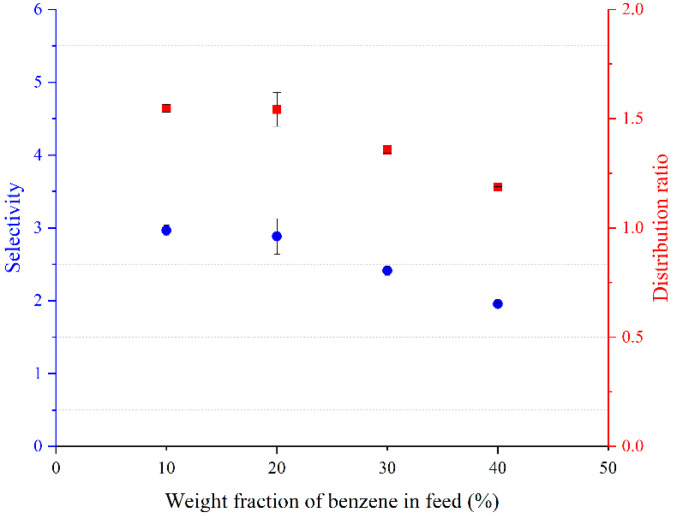
Variation of the distribution ratio and selectivity with benzene weight fraction in the feed for [Benzene (1) + Cyclohexane (2) + BTBACl:Cre (1:2) (3)].

**Figure 7:**
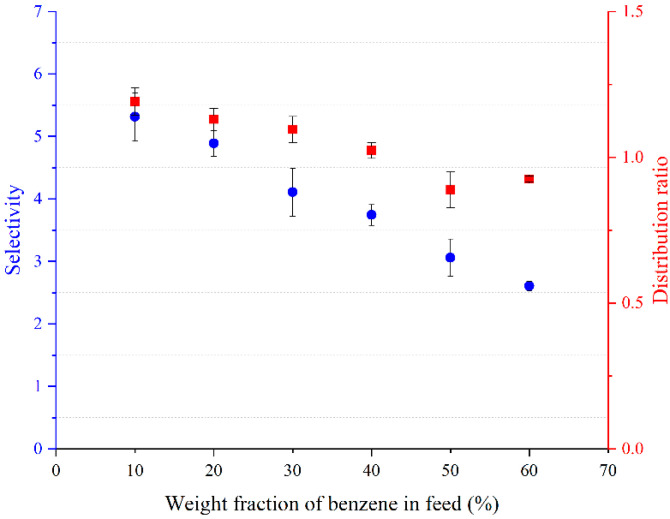
Variation of the distribution ratio and selectivity with benzene weight fraction in the feed for [Benzene (1) + Cyclohexane (2) + TBPB:PTSA (1:1) (3)].

The distribution coefficients and selectivity using BTBACl:Ph (1:2), BTBACl:Cre (1:2), and TBPB:PTSA (1:1) are shown in Figure 5, Figure 6 and Figure 7, respectively. It can be observed that the weight fraction of benzene in the feed does not have much effect on the distribution ratio of BTBACl:Ph (1:2) and BTBACl:Cre (1:2), as only a slight change in the distribution ratio was observed at higher benzene concentrations in the feed in both ternary systems. This suggests that the extraction capacity of benzene from benzene-cyclohexane mixtures using BTBACl:Ph (1:2) and BTBACl:Cre (1:2) does not change significantly at different benzene concentrations in the feed. This would open a wide applicability of both ESs as extractants for higher benzene concentrations in the feed. In contrast, when TBPB:PTSA (1:1) was used as the extractant, the distribution ratio was less than one when the benzene concentration in the feed was greater than 40 wt%. This explains the change in the slope of tie lines from positive to negative values in Figure 4c, where the negative slopes indicate a higher amount of benzene in the raffinate phase than in the extract phase. On the other hand, it is observed that all the selectivity values for all the ternary systems are greater than one, indicating the possibility of separation by liquid–liquid extraction.

The Othmer-Tobias (Equation (4)) [45] and Hand correlations (Equation (5)) [46] were employed to all ternary systems in order to determine the reliability of the experimental LLE data:

(4) $$\ln\left(\frac{1-w_{\mathrm{cyc}}^{\prime \prime }}{w_{\mathrm{cyc}}^{\prime \prime }}\right)=a+b\ln\left(\frac{1-w_{\mathrm{ES}}^{\prime }}{w_{\mathrm{ES}}^{\prime }}\right)$$

(5) $$\ln\left(\frac{w_{\mathrm{Ben}}^{\prime \prime }}{w_{\mathrm{cyc}}^{\prime \prime }}\right)=c+d\ln\left(\frac{w_{\mathrm{Ben}}^{\prime }}{w_{\mathrm{ES}}^{\prime }}\right)$$

where *w*_cyc_, *w*_ES_, and *w*_Ben_ denote the concentrations of cyclohexane, ES, and benzene, respectively, a and b refer to the fitting parameters of the Othmer-Tobias correlation, c and d are the fitting parameters of the Hand correlation, and superscripts ′ and ″ refer to the bottom and extract phases, respectively. Table 2 shows the parameters of the Othmer-Tobias and Hand equations. The degree of accuracy of the LLE experimental results is demonstrated by the linearity of the plot (regression coefficient R^2^ is close to unity).

### 2.3. Comparison with Other Solvents

**Figure 8:**
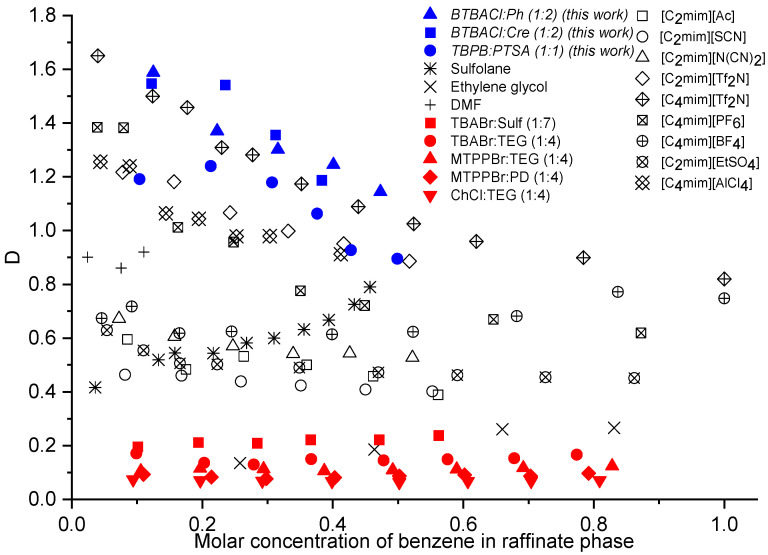
Distribution ratio of organic solvents, ILs, and ESs for the extractive separation of benzene and cyclohexane.

**Figure 9:**
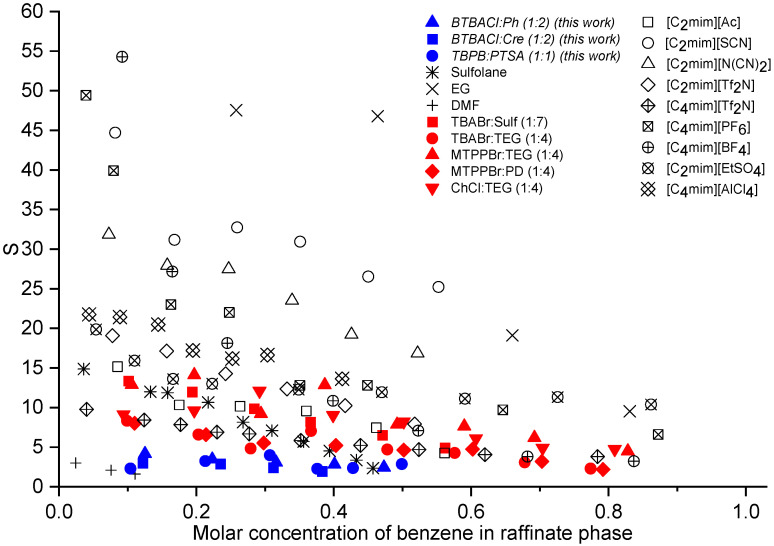
Selectivity of organic solvents, ILs, and ESs for the extractive separation of benzene and cyclohexane.

The extraction process efficiency can be evaluated by the distribution ratio of benzene and the selectivity of ES. In Figure 8 and Figure 9, the values from this work are compared with some previous reports using different types of extraction solvents, namely organic solvents [47,48], ILs [41,49,50,51], and ESs [26]. Sulfolane was used as a benchmark that represents the extractive performance of organic solvents used in the industry. In addition, organic solvents with high selectivity (ethylene glycol, EG) and distribution ratio (dimethylformamide, DMF) were also used for comparison [47]. The full data of both plots and their corresponding ternary molar compositons are summarized in Table S9 of the Supporting Information.

As can be seen in Figure 8, BTBACl:Ph (1:2) and BTBACl:Cre (1:2) demonstrated the highest benzene distribution ratio compared to other solvents, regardless of the type of solvent, i.e., ESs, ILs, or conventional solvents. For instance, comparing only the perspective of ES, when the molar concentration of benzene was around 0.1, the benzene distribution ratio was on the order of BTBACl:Ph > BTBACl:Cre > TBPB:PTSA (1:1) > > TBABr:Sulf (1:7) > TBABr: TEG (1:4) > MTPPBr: TEG (1:4) > MTPPBr: PD (1:4) > ChCl: TEG (1:4). This demonstrates the potential of the three ESs as green solvent alternatives for the separation of benzene and cyclohexane. As for the selectivity depicted in Figure 9, although all ESs showed the lowest values, all values were greater than unity, indicating the feasibility of the extraction process. The three ESs also showed higher selectivity than the common organic solvent DMF. Based on the values of benzene distribution ratio and the selectivity, it can be observed that the performance of ESs to separate the mixture of benzene and cyclohexane was generally lower than those given by ILs. Nonetheless, this finding does not disregard the feasibility of using ESs for such separation as their experimental selectivity values were higher than unity, indicating the possibility of the extraction process. Furthermore, ESs can also be regenerated and recycled back into the feed stream after the extracted benzene and cyclohexane are removed by anti-solvent extraction or by distillation. In the perspective of extractive separation performance, it can be concluded that ILs are more superior; however, ESs offer remarkable advantages in another perspectives, i.e., cheaper, greener, and easier to prepare than ILs.

## 3. Materials and Methods

### 3.1. Molecular Geometry Optimization

The geometry optimization of the species involved was conducted using the TMole-X programme package. After the chemical structure of the target molecule was drawn, the geometry optimization was performed at the Hartree-Fock level and the 6-31G* basis set. The .cosmo file was then generated by a single-point calculation using DFT with Becke-Perdew and the Triple-ζ Zeta Valence Potential (TZVP) basis set. Finally, the COSMOthermX programme was used to import the .cosmo files with the parameterization of BP _TZVP_C30_1301.ctd.

### 3.2. List of ESs for COSMO-RS Screening

The COSMO-RS screening was performed by collecting 20 ESs with aromatic structure from the literature in addition to three new ESs proposed in this work. The studied ESs are shown in Table 3.

### 3.3. ES Representation in COSMOtherm-X

Since a single ES is composed of more than one molecule, employing its representation method in the COSMOtherm-X programme is crucial. ES representation follows the same approach as for ILs, which can be selected from three approaches: (i) the metafile, (ii) the ion-pair, and (iii) the electroneutral. For ILs, the electroneutral approach was usually chosen because it is the most suitable and closest to the actual nature of ILs, where ions are treated as two different compounds in an equimolar mixture. Similarly, this approach was adopted in this study to represent ESs in COSMO-RS based on the molar composition of their constituents (salt cation, salt anion, and hydrogen bond donor (HBD)). The mathematical adaptation has been described in detail in our previous work [35,36,37].

### 3.4. Selectivity, Capacity, and Performance Index

The activity coefficient at infinite dilution $\gamma^{\infty }$ can be adopted to evaluate the maximum capacity and selectivity of targeted ESs. The value of $\gamma^{\infty }$ describes the solute–solvent interactions at an infinitesimal concentration of the solute. In other words, the interaction is evaluated at the far-reaching concentration of the solvent as the concentration of the solute approaches zero. Since $\gamma^{\infty }$ accounts only for the IL–solute interaction behaviour and does not reflect the exact extraction ability, it can be further used for calculating the selectivity (*S*∞) and capacity (*C*∞) of an ES at infinite dilution. S∞ defines the ability of an ES to interact more with either one of the compounds, and less with another. Therefore, in this case, the selectivity of an ES towards benzene in comparison with cyclohexane ($S_{B,C}^{\infty }$) can be expressed in terms of the ratio of the activity coefficient for cyclohexane to benzene. This means an ES with high selectivity towards benzene has a high value of $\gamma_{C}^{\infty }$ and a low value of $\gamma_{B}^{\infty }$.

(6) $$S_{B,C}^{\infty }=\frac{\gamma_{C}^{\infty }}{\gamma_{B}^{\infty }}$$

In addition, the amount of an ES required for the extraction process can also be qualitatively determined from the value of *C*∞. The capacity of an ES for benzene ($C_{B}^{\infty })$ indicates the maximum amount of benzene that can be dissolved in the ES, which can be calculated with the inverse of the activity coefficient for benzene:

(7) $$C_{B}^{\infty }=\frac{1}{\gamma_{B}^{\infty }}$$

The final parameter for evaluating the solvent feature in this extraction process is the performance index (PI). It combines both features of capacity and selectivity for estimating the overall performance of an ES. The PI is simply expressed as the product of $S_{B,C}^{\infty }$ and $C_{B}^{\infty }$:

(8) $$\mathrm{PI}=S_{B,C}^{\infty }\times C_{B}^{\infty }$$

Table 4 provides a list of the compounds that were used in this work. These chemicals were used without further purification due to their high purity. For each ES, the HBD was combined with the salt in screw cap vials. The vials were then shaken with incubation shakers. Shaking was performed at a speed of 200 rpm and a temperature of 100 °C until a clear liquid was obtained. 

### 3.5. Liquid–Liquid Equilibria (Lle) Measurements

The LLE data of the ternary mixtures [benzene + cyclohexane + ES] were measured experimentally at 298.15 K. Three ESs were selected for the measurement of experimental LLE data, namely BTBACl:Ph (1:2), BTBACl:Cre (1:2), and TBPB:PTSA (1:1). These ESs were selected mainly because of their liquid nature at room temperature. The desired quantities of ES, benzene, and cyclohexane were mixed in tightly sealed plastic vials within their immiscible concentration range to perform LLE experiments and to achieve tie lines for each system. The feed mixture was obtained by mixing the weighed chemicals with an analytical balance (±0.0001 g). The vials were then placed in a shaking incubator to control the shaking rate at 200 RPM and the temperature at 298.15 K. The mixture was stirred for 4 h to ensure adequate mixing between the two layers, i.e., extract and raffinate. The vials were then left to rest in the incubator for 12 h to ensure that the equilibrium state was fully reached. Once equilibrium was reached, samples were collected from both layers and analyzed by gas chromatography (GC).

To obtain tie lines, a Trace GC ultra (from Thermo Scientific) was used to determine the compositions of the ES phase and the extract phase (hydrocarbon-rich layer). The chromatograph was equipped with an Rtx-1 column (30 m × 0.25 mm × 0.25 μm) and a flame ionization detector (FID). The temperature ramp of the GC was set to 358.2 K at a rate of 10 K/min. The temperatures for injection and FID were maintained at 584 K. The column oven was maintained at 308.2 K for 2 min. Helium at a constant flow rate of 30 mL/min was used as the carrier gas. A split ratio of 16 was considered with an injection volume of 0.3 µL. Acetonitrile was used as a diluent. Using this method, the compositions of benzene and cyclohexane in each layer were measured and the corresponding compositions of ES in each layer were determined by mass balance calculations. ES was considered as a single pseudo-component to calculate mole fractions, with a mixture containing benzene and cyclohexane. A calibration curve for benzene and cyclohexane was constructed to measure the composition. Each measurement was tripled by GC and the molar composition was estimated to be ±0.009. To confirm the absence of ES in the hydrocarbon-rich phase, ^1^H NMR was performed. A JEOL RESPNANCE spectrometer (ECX-500 II) was used to record the ^1^H NMR spectra at 298.15 K and CDCl_3_ was used as solvent. 

In addition, Fourier transform infrared (FTIR) analysis was performed to confirm the structure of the ESs. For example, Figure S4 shows that in pure PTSA, the peaks representing the symmetric and asymmetric stretching of SO_3_ in the range of 2800–1800 cm^−1^ disappeared after the formation of ES. It can be seen that the hydrogen bonding occurred between PTSA and TBPB instead of between PTSA and H_2_O. Similar phenomena of disappearance of the SO_3_ peak to form a bond for the components of ES were also previously [62,63] reported in PTSA-based ESs, e.g., PTSA:tetrabutylphosphonium chloride (1:1), PTSA:tetrabutylammonium chloride (1:1), and PTSA:ChCl (1:1). Hydrogen bond formation between BTBACl and m-cresol is the driving force behind the synthesis of ES. As shown in Figure S5, absorptions associated with O-H at 3336 cm^−1^ were observed in the FTIR spectra of pure m-cresol. The O-H vibration of m-cresol shifted to 3172 cm^−1^ in the FTIR of ES. These shifts might have been caused by the transfer of an electron from an oxygen atom to the hydrogen bond, leading to a decrease in the force constant [64,65]. When the ES was formed, the shift in O-H vibrations indicated hydrogen bonding between BTBACl and m-cresol. In Figure S6, FTIR analysis of pure phenol showed the formation of O-H (3243 cm^−1^), C=C (1475 and 1596 cm^−1^), and C-O (1233 cm^−1^). In the FTIR spectrum of ES, all the peaks of phenol were approximately in the same range as the peaks of pure phenol, except for the peak associated with the O-H bond. In the ES, the O-H vibrations of pure phenol (3243 cm^−1^) shifted to 3169 cm^−1^. This phenomenon may indicate the sharing of oxygen atom electrons to form the hydrogen bond between phenol and BTBACl during the formation of ES. Similar behavior was observed by Mehran et al. [66] for phenol-based ESs.

## 4. Conclusions

The potential of ESs containing constituents with an aromatic structure for extractive separation of benzene–cyclohexane mixture was explored in this work. COSMO-RS was used to preselect the ESs. From the 23 candidates screened, 3 ESs were selected for experimental validation, where their extractive performances to separate benzene and cyclohexane mixture were studied through liquid–liquid extraction process. Ternary LLE data were acquired for the three ESs, i.e., BTBACl:Ph (1:2)/BTBACl:Cre (1:2)/TBPB:PTSA (1:1) + benzene + cyclohexane at 25 °C and 101.325 kPa. It was found that BTBACl:Cres (1:2) and BTBACl:Ph (1:2) show high distribution compared to other solvents, i.e., conventional solvents, ILs, and other ESs in previous works.

## Figures and Tables

**Table 1:** Molar composition of experimental tie-lines, distribution ratio (D), and selectivity (S) data for three systems at 101.325 kPa and T = 298.15 K.

Raffinate Phase		Extract Phase				D_Ch_	D_Ben_	S
*x*_Ben_	*x*_Ch_	*x*_Ben_	*x*_Ch_	*x*_HBA_	*x*_HBD_			
Benzene (1) + Cyclohexane (2) + BTBACl (3) + Ph (4) (1:2, HBA:HBD molar ratio)								
0.079	0.921	0.125	0.350	0.175	0.349	0.381	1.588	4.17
0.163	0.837	0.223	0.332	0.148	0.297	0.396	1.370	3.46
0.243	0.757	0.316	0.320	0.121	0.243	0.423	1.301	3.08
0.322	0.678	0.401	0.299	0.100	0.200	0.441	1.245	2.82
0.413	0.587	0.473	0.275	0.084	0.168	0.468	1.144	2.44
Benzene (1) + Cyclohexane (2) + BTBACl (3) + Cre (4) (1:2, HBA:HBD molar ratio)								
0.079	0.921	0.122	0.480	0.133	0.265	0.521	1.547	2.97
0.153	0.847	0.235	0.453	0.104	0.208	0.535	1.542	2.88
0.230	0.770	0.312	0.432	0.085	0.170	0.562	1.356	2.41
0.323	0.677	0.383	0.411	0.069	0.137	0.607	1.187	1.95
Benzene (1) + Cyclohexane (2) + TBPB (3) + PTSA (4) (1:1, HBA:HBD molar artio)								
0.089	0.911	0.106	0.204	0.345	0.345	0.518	1.191	5.31
0.179	0.821	0.202	0.190	0.304	0.304	0.381	1.130	4.89
0.267	0.733	0.293	0.195	0.256	0.256	0.296	1.095	4.10
0.359	0.641	0.368	0.175	0.229	0.229	0.469	1.023	3.74
0.461	0.539	0.409	0.157	0.217	0.217	0.391	0.888	3.06
0.553	0.447	0.512	0.159	0.165	0.165	0.312	0.926	2.61

Standard uncertainties are: u(T) = 0.1 K, u(P) = 0.1 kPa, u(x) = 0.009.

**Table 2:** Parameters of Othmer-Tobias and Hand correlation for ternary systems [benzene + cyclohexane + BTBACl:Ph] and [benzene + cyclohexane + BTBACl:Cre].

ES	Othmer-Tobias			Hand		
	a	b	*R* ^2^	c	d	*R* ^2^
BTBACl:Ph (1:2)	0.929	1.753	0.982	0.794	1.004	0.998
BTBACl:Cre (1:2)	0.194	1.881	0.998	0.356	0.947	0.994
TBPB:PTSA (1:1)	2.363	1.749	0.974	1.810	1.124	0.988

**Table 3:** List of ESs screened in this work using COSMO-RS with their abbreviations.

No	HBA	HBD	Ratio	Abbreviation	Ref.
1	Choline chloride	Benzoic acid	1:1	ChCl:BZA (1:1)	[52,53,54]
2	Choline chloride	Benzamide	1:2	ChCl:BZM (1:2)	[55]
3	Choline chloride	p-toluenesulfonic acid	1:2	ChCl:PTSA (1:2)	[56]
4	Tetrabutylammonium chloride	p-toluenesulfonic acid	1:2	TBACl:PTSA (1:2)	[56]
5	Tetrabutylammonium bromide	p-toluenesulfonic acid	1:2	TBABr:PTSA (1:2)	[56]
6	Tetraethylammonium chloride	p-toluenesulfonic acid	1:2	TEACl:PTSA (1:2)	[56]
7	Tetraethylammonium bromide	p-toluenesulfonic acid	1:2	TEABr:PTSA (1:2)	[56]
8	Choline chloride	p-aminosalicylic acid	1:2	ChCl:PAS (1:2)	[56]
9	Trimethylglycine	Benzoic acid	1:1.5	TMGly:BZA (1:1.5)	[57]
10	Trimethylglycine	Salicylic acid	1:1.5	TMGly:SA (1:1.5)	[57]
11	Trimethylglycine	4-chlorobenzoic acid	1:1.5	TMGly:4-CBZ (1:1.5)	[57]
12	Trimethylglycine	3-chlorobenzoic acid	1:1.5	TMGly:3-CBZ (1:1.5)	[57]
13	Trimethylglycine	2-chlorobenzoic acid	1:1.5	TMGly:2-CBZ (1:1.5)	[57]
14	Trimethylglycine	2-furoic acid	1:2	TMGly:2-FA (1:2)	[57]
15	Benzyltrimethylammonium chloride	p-toluenesulfonic acid	3:7	BTMACl:PTSA (3:7)	[58]
16	Benzyltrimethylammonium chloride	Oxalic acid	1:1	BTMACl:OA (1:1)	[58]
17	Benzyltrimethylammonium chloride	Citric acid	1:1	BTMACl:CA (1:1)	[58]
18	N,N-diethylenethanolammonium Cl	p-toluenesulfonic acid	1:3	DEEACl:PTSA (1:3)	[59]
19	Allyltriphenylphosphonium Br	p-toluenesulfonic acid	1:3	ATPPBr:PTSA (1:3)	[60]
20	Choline chloride	p-toluenesulfonic acid	1:2	ChCl:PTSA (1:2)	[61]
21	Benzyltributylammonium chloride	Phenol	1:2	BTBACl:Ph (1:2)	*
22	Benzyltributylammonium chloride	m-Cresol	1:2	BTBACl:Cre (1:2)	*
23	Tetrabutylphosphonium bromide	p-toluenesulfonic acid	1:1	TBPB:PTSA (1:1)	*

* This work.

**Table 4:** List of chemicals used in the experimental work.

Chemical	Formula	Purity (wt%)	Supplier	Country
Benzene	C_6_H_6_	99.5	Panreac	Spain
Cyclohexane	C_6_H_12_	99.5	Analar	England
BTBACl	C_6_H_5_CH_2_N(Cl)(CH_2_CH_2_CH_2_CH_3_)_3_	97	Aldrich	Netherland
Phenol	C_6_H_6_O	99.5	VWR International	Belgium
m-Cresol	C_7_H_8_O	99	Scharlau	Spain
TBPB	(CH_3_CH_2_CH_2_CH_2_)4P(Br)	98	Aldrich	China
PTSA	C_7_H_8_O_3_S	98.5	Sigma-Aldrich	Japan
Deuterated Chloroform	CDCl_3_	99.8	Sigma-Aldrich	German
Acetonitrile	C_2_H_3_N	99.9	VWR International	England

The structures of the studied chemicals are presented in Table S2.

## Supplementary Materials

The following supporting information can be downloaded at: https://www.mdpi.com/article/10.3390/molecules27134041/s1, Figure S1: CDCl3: (a) pure BTBACl:Ph (1:2) ES; (b) Extract layer. Figure S2: 1H NMR spectra in CDCl3: (a) pure BTBACl:Cre (1:2) ES; (b) Extract layer. Figure S3: 1H NMR spectra in CDCl3: (a) pure TBPB:PTSA (1:1) ES; (b) Extract layer. Figures S4–S6: FTIR analysis of all three ESs and their individual componenets. Table S1: Some examples of ESs in various fields. Table S2: Structures of the studied chemicals. Tables S3 and S4: Standard deviation (STDEV) on measured solubilities with BTBACl:Ph (1:2) (3) for the top layer and bottom layer. Tables S5 and S6: STDEV on measured solubilities with BTBACl:Cre (1:2) (3) for the top layer and bottom layer. Tables S7 and S8: Standard deviation (STDEV) on measured solubilities with TBPB:PTSA (1:1) (3) for the top layer and bottom layer. Table S9: Molar ternary compositions, benzene distribution ratio (DBz) and solvent selectivity (S) from previous works involving organic solvents, ILs, and ESs. x1, x2, and x3 represents the molar composition of benzene, cyclohexane, and solvents, respectively. References [67,68,69,70,71,72,73,74,75,76,77,78,79,80,81,82,83,84,85,86,87,88,89,90,91,92,93,94,95,96,97,98] are cited in the supplementary materials.

## Sample Availability

Samples of the compounds are not available from the authors.
