# Supplementary material for: Separation of Benzene and Cyclohexane Using Eutectic Solvents with Aromatic Structure
Source: Molecules. 2022 Jun 23;27(13):4041. doi: 10.3390/molecules27134041 (PMC9268653; doi:10.3390/molecules27134041)
Supplement: Supplementary file 1 [file molecules-27-04041-s001.zip › molecules-1737849-supplementary.pdf]

**Supporting Information for publication**

# Separation of benzene and cyclohexane using deep eutectic solvents with aromatic structure

Mohamed K. Hadj-Kali[1,*], M. Zulhaziman M. Salleh[2,*], Irfan Wazeer[1], Ahmad Alhadid[3], Sarwono Mulyono[1]

[1] Chemical Engineering Department, King Saud University, P.O. Box 800, Riyadh 11421, Saudi Arabia
[2] Department of Chemical and Process Engineering, Faculty of Engineering and Built Environment, 43600 Bangi, Selangor, Malaysia
[3] Biothermodynamics, TUM School of Life Sciences, Technical University of Munich, Maximus-von-Imhof-Forum 2, D-85354 Freising, Germany

*Corresponding Authors: mhadjkali@ksu.edu.sa; zulhaziman@ukm.edu.my

## List of figures

Figure S1. $^1$H NMR spectra in $CDCl_3$: (a) pure BTBACl:Ph (1:2) DES; (b) top layer.

Figure S2. $^1$H NMR spectra in $CDCl_3$: (a) pure BTBACl:Cre (1:2) DES; (b) top layer.

Figure S3. $^1$H NMR spectra in CDCl3: (a) pure TBPB:PTSA (1:1) DES; (b) top layer.

Figure S4: FTIR analysis for DES TBPB:PTSA (1:1). Red line indicates the tetrabutylphosphonium salt, blue indicates p-toluenesulfonic acid and black indicates the DES.

Figure S5: FTIR analysis for DES BTBACl:m-cresol (1:2). Red line indicates benzyltributylammonium chloride, blue indicates m-cresol and black indicates the DES.

Figure S6: FTIR analysis for DES BTBACl:phenol (1:2). Red line indicates benzyltributylammonium chloride, blue indicates phenol and black indicates the DES.

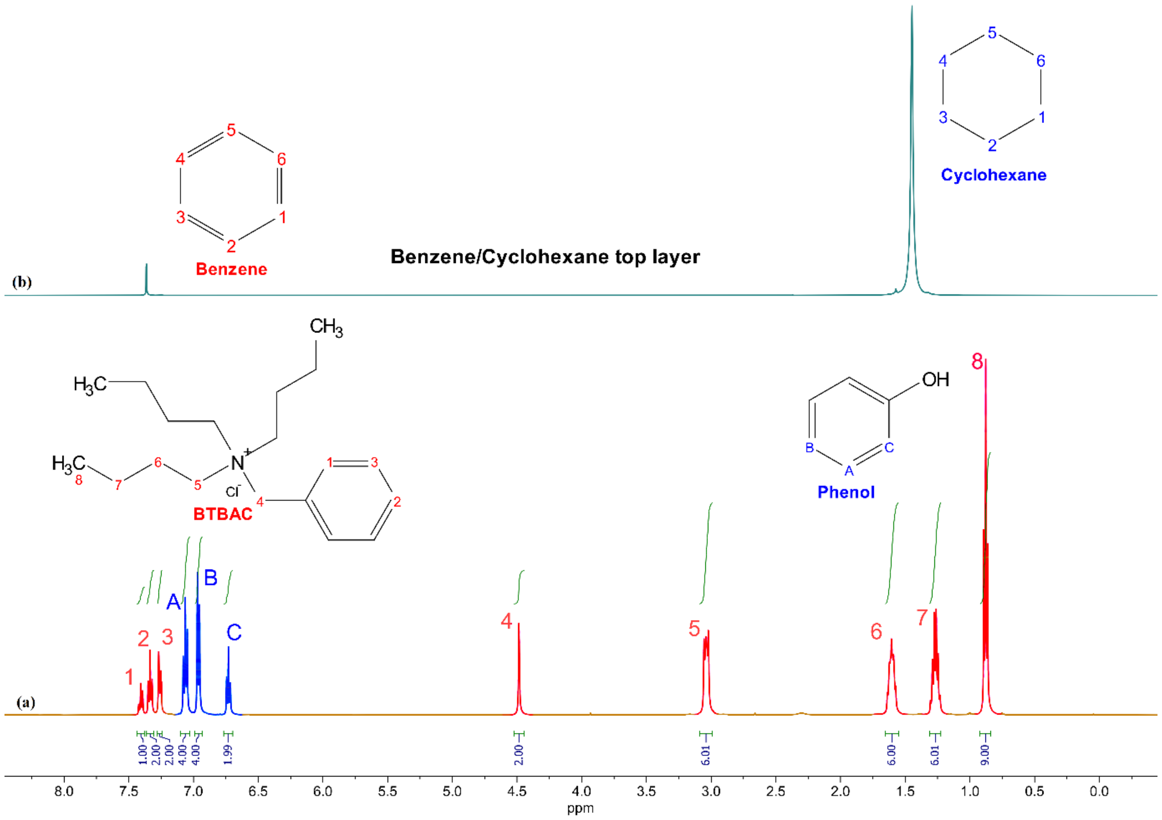


Figure S1. $^1$H NMR spectra in $CDCl_3$: (a) pure BTBACl:Ph (1:2) DES; (b) top layer.

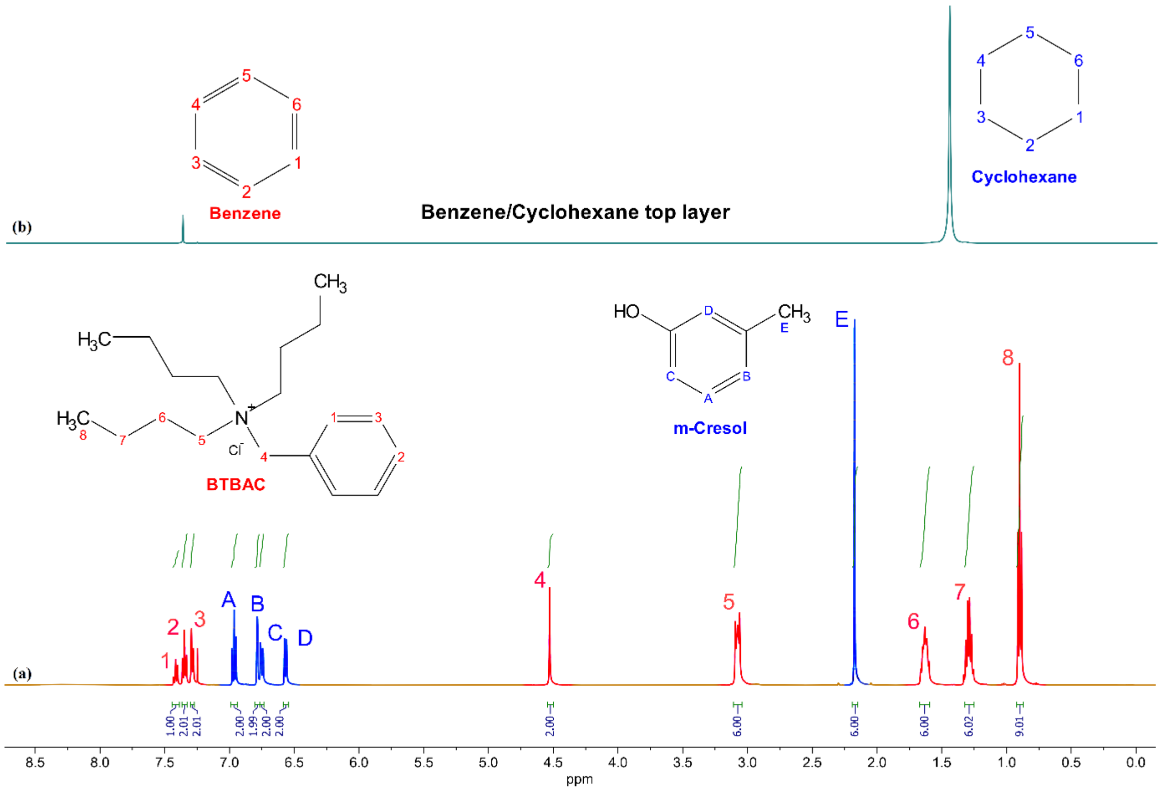


Figure S2. $^1$H NMR spectra in $CDCl_3$: (a) pure BTBACl:Cre (1:2) DES; (b) top layer.

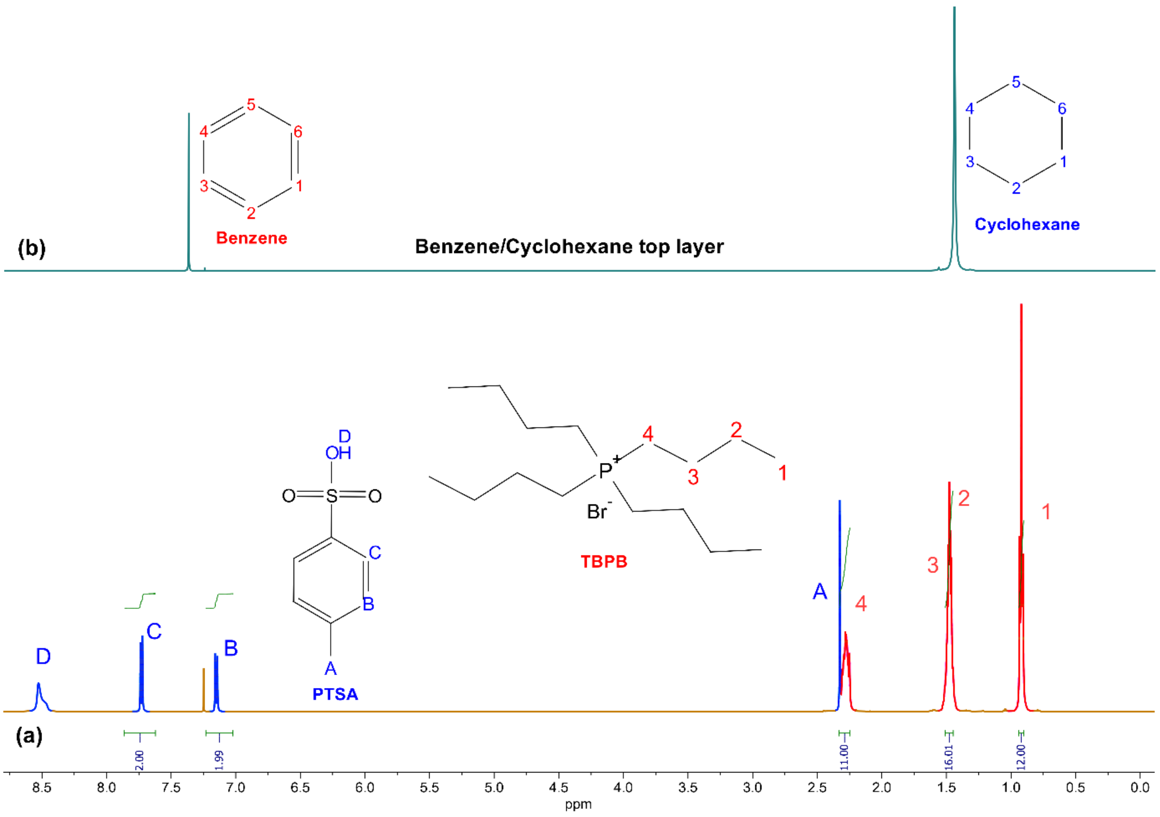


Figure S3. $^{1}$H NMR spectra in $CDCl_3$: (a) pure TBPB:PTSA (1:1) DES; (b) top layer.

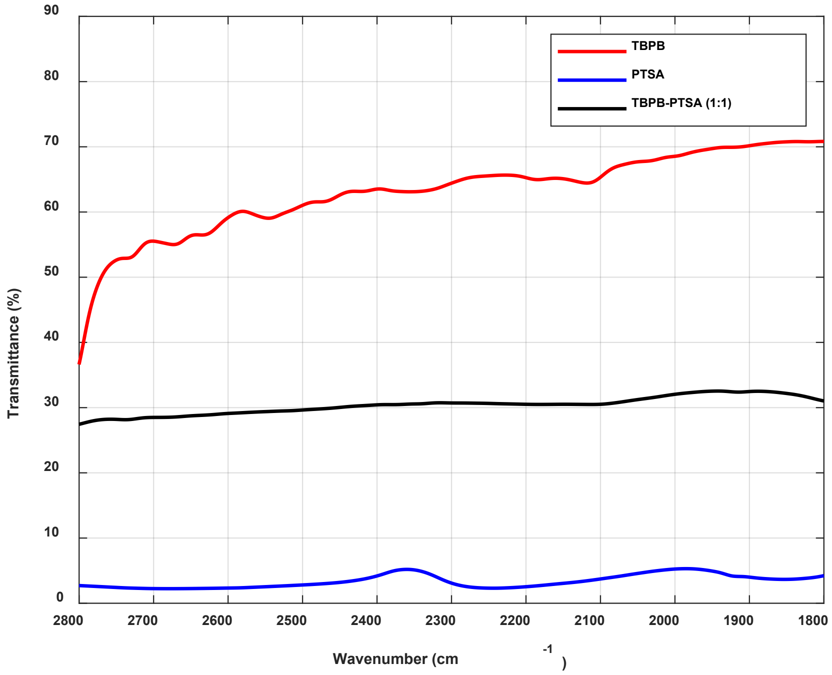


Figure S4: FTIR analysis for DES TBPB:PTSA (1:1). Red line indicates the tetrabutylphosphonium salt, blue indicates p-toluenesulfonic acid and black indicates the DES.

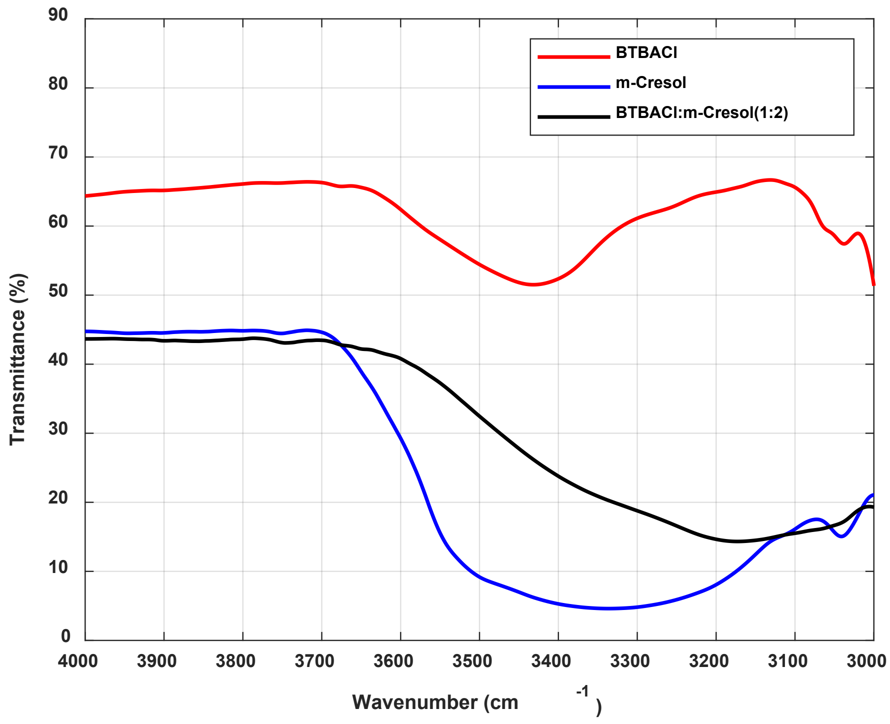


Figure S5: FTIR analysis for DES BTBACl:m-cresol (1:2). Red line indicates benzyltributylammonium chloride, blue indicates m-cresol and black indicates the DES.

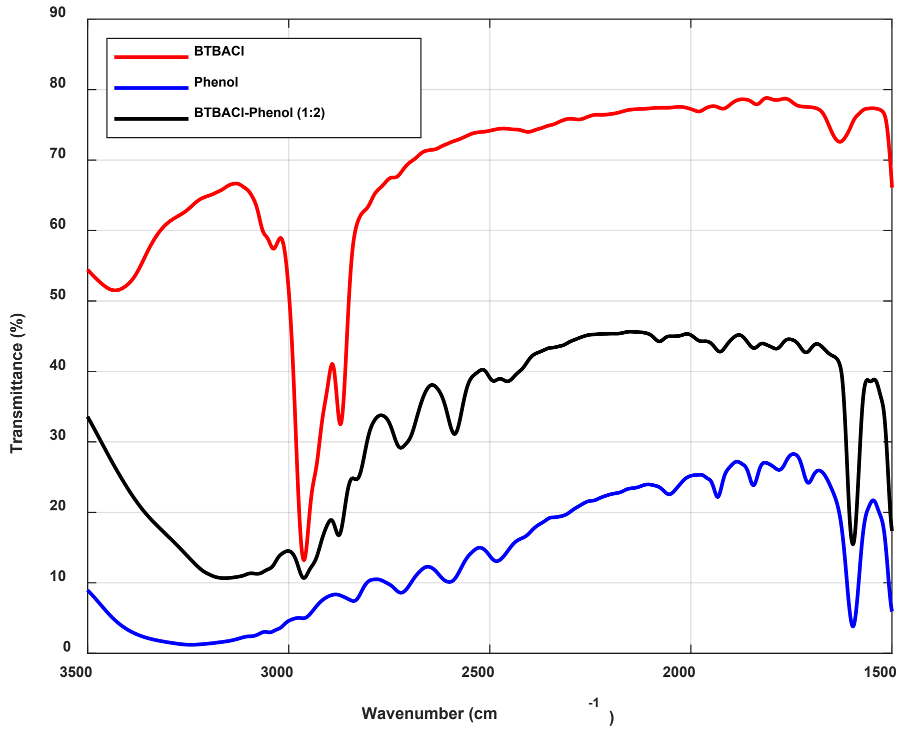


Figure S6: FTIR analysis for DES BTBACl:phenol (1:2). Red line indicates benzyltributylammonium chloride, blue indicates phenol and black indicates the DES.

## List of tables

Table S1. Some examples of DESs applications in various fields.

Table S2. Structures of the studied chemicals.

Table S3: Standard deviation (STDEV) on measured solubilities of Benzene (1) / Cyclohexane (2) mixture with BTBACl:Phenol (1:2) (3) for mole fractions x: Top layer.

Table S4: STDEV on measured solubilities of Benzene (1) / Cyclohexane (2) mixture with BTBACl:Ph (1:2) (3) for mole fractions x: Bottom layer.

Table S5: STDEV on measured solubilities of Benzene (1) / Cyclohexane (2) mixture with BTBACl:Cre (1:2) (3) for mole fractions x: Top layer.

Table S6: STDEV on measured solubilities of Benzene (1) / Cyclohexane (2) mixture with BTBACl:Cre (1:2) (3) for mole fractions x: Bottom layer.

Table S7: STDEV on measured solubilities of Benzene (1) / Cyclohexane (2) mixture with TBPB:PTSA (1:1) (3) for mole fractions x: Top layer.

Table S8: STDEV on measured solubilities of Benzene (1) / Cyclohexane (2) mixture with TBPB:PTSA (1:1) (3) for mole fractions x: Bottom layer.

Table S9: Molar ternary compositions, benzene distribution ratio ($D_{Bz}$) and solvent selectivity (S) from previous works involving organic solvents, ILs and DESs. $x_1$, $x_2$ and $x_3$ represents the molar composition of benzene, cyclohexane and solvents, respectively.

Table S1. Some examples of DESs applications in various fields.

| Application | Analyte/system | DESs | Refs. |
|---|---|---|---|
| **Separation and $CO_2$ capture** | | | |
| Liquid-liquid extraction | Heptane/ethaline mixture, removal of volatile fatty acids from aqueous solutions, removal of pertechnate in aqueous media | ChCl[1]:glycerol (1:2), ChCl: levulinic acid (1:2), ChCl:EG[2] (1:2), MTOAC[3]:DA[4] (1:2), MTOB[5]: DA (1:2) | [1-3] |
| Metal extraction | Removal of various metals including indium, boron, copper, cobalt, manganese, cobalt, nickel and lithium | DA:lidocaine (2:1), thymol: 1-decanol (2:1), thymol:MPD[6] (2:1), menthol: 1-decanol (2:1), menthol:MPD (2:1) | [4-7] |
| $CO_2$ capture | solubilization of $CO_2$ | Betaine:lactic acid (1:1), ChCl:guaiacol (1:1), MTPPB[7]/glycerol (1:3), ChCl:urea (1:2), ChCl:EG (1:2) | [8-12] |
| **Electrodeposition** | | | |
| ionometallurgy | Recovery of gold, tellurium, sulphides and tellurides | ChCl:EG (1:2) | [13] |
| electrodeposition | electrodeposition of zinc, tin, indium, cobalt, nickel and Samarium | ChCl:EG (1:2), ChCl:urea (1:2) | [14-19] |
| metal oxides solubility | Various metal oxides | ChCl:EG (1:2), ChCl:urea (1:2), ChCl:malonic acid (1:1) | [20] |
| **Nanotechnology** | | | |
| Synthesis of nanoparticles | Gold nanoparticles | ChCl:urea (1:2) | [21] |
| Synthesis of nanoparticles | CuCl nanoparticles | ChCl:urea (1:2) | [22] |
| Synthesis of nanocrystals | Silver nanocrystals | silver triflate:acetamide (1:4) | [23] |
| **Organic chemistry** | | | |
| Biochemistry | Glycerol extraction from biodiesel | ChCl:glycerol (1:2), MTPPB:EG (1:3) | [24, 25] |
| Enzymatic biodiesel production | Triglyceride conversion of miglyol oil | ChCl:urea (1:2), ChCl:glycerol (1:2) | [26, 27] |
| **Solar energy and battery technology** | | | |
| Vanadium−iron redox flow battery testing | Solubility of vanadium and iron | ChCl:urea (1:2), ChCl:EG (1:2) | [28] |
| Solar energy | Solar cell | ChCl:glycerol (1:2) | [29, 30] |
| **Electrochemical sensor platform** | | | |
| Electrochemical sensor | Synthesis of nanoporous gold | ChCl:urea (1:2) | [31] |

| | | | |
|---|---|---|---|
| Electrochemical sensor | Determination of nitrofurazone | ChCl:urea:glycerol (1:1:1) | [32] |
| **Photosynthesis** | | | |
| Photosynthesis | Photosynthetic reaction centers of rhodobacter sphaeroides | ChCl:EG (1:3), ChCl:malonic acid (1:1) | [33] |

[1]Choline chloride,[2]ethylene glycol, [3]methyltrioctylammonium chloride, [4]decanoic acid, [5]methyltrioctylammonium bromide, [6]2-methyl 2,4-pentanediol, [7]methyltriphenylphosphonium bromide

Table S2. Structures of the studied chemicals.

| | |
|---|---|
| Benzene | 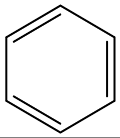 |
| Cyclohexane | 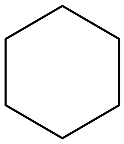 |
| BTBACl | 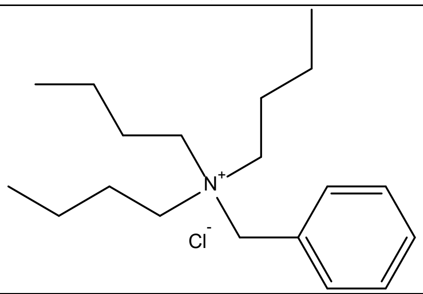 |
| Phenol | 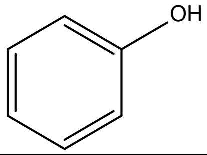 |
| m-Cresol | 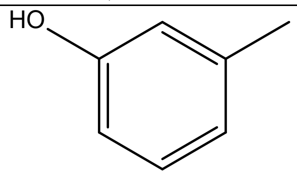 |
| TBPB | 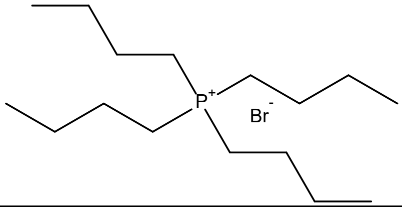 |
| PTSA | 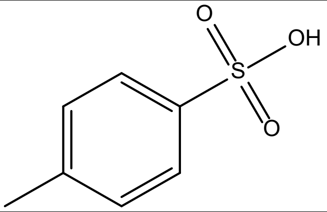 |

Table S3: Standard deviation (STDEV) on measured solubilities of Benzene (1) / Cyclohexane (2) mixture with BTBACl:Ph (1:2) (3) for mole fractions x: Top layer

| **Mole fraction** | | | **Average mole fraction** | | | **STDEV mole fraction** | | |
|---|---|---|---|---|---|---|---|---|
| $x_1$ | $x_2$ | $x_3$ | $x_1$ | $x_2$ | $x_3$ | $x_1$ | $x_2$ | $x_3$ |
| 0.0784 | 0.9216 | 0 | 0.0789 | 0.9211 | 0 | 0.0005 | 0.0005 | 0 |
| 0.0793 | 0.9207 | 0 | | | | | | |
| 0.0791 | 0.9209 | 0 | | | | | | |
| 0.1665 | 0.8335 | 0 | 0.1631 | 0.8369 | 0 | 0.0048 | 0.0048 | 0 |
| 0.1598 | 0.8402 | 0 | | | | | | |
| 0.2450 | 0.7550 | 0 | 0.2429 | 0.7571 | 0 | 0.0018 | 0.0018 | 0 |
| 0.2422 | 0.7578 | 0 | | | | | | |
| 0.2416 | 0.7584 | 0 | | | | | | |
| 0.3230 | 0.6770 | 0 | 0.3225 | 0.6775 | 0 | 0.0005 | 0.0005 | 0 |
| 0.3220 | 0.6780 | 0 | | | | | | |
| 0.3224 | 0.6776 | 0 | | | | | | |
| 0.4181 | 0.5819 | 0 | 0.4134 | 0.5866 | 0 | 0.0041 | 0.0041 | 0 |
| 0.4114 | 0.5886 | 0 | | | | | | |
| 0.4107 | 0.5893 | 0 | | | | | | |

Table S4: STDEV on measured solubilities of Benzene (1) / Cyclohexane (2) mixture with BTBACl:Ph (1:2) (3) for mole fractions x: Bottom layer

| **Mole fraction** | | | **Average mole fraction** | | | **STDEV mole fraction** | | |
|---|---|---|---|---|---|---|---|---|
| $x_1$ | $x_2$ | $x_3$ | $x_1$ | $x_2$ | $x_3$ | $x_1$ | $x_2$ | $x_3$ |
| 0.1265 | 0.3524 | 0.5211 | 0.1254 | 0.3505 | 0.5241 | 0.0012 | 0.0046 | 0.0045 |
| 0.1242 | 0.3538 | 0.5220 | | | | | | |
| 0.1254 | 0.3452 | 0.5294 | | | | | | |
| 0.2159 | 0.3417 | 0.4424 | 0.2235 | 0.3317 | 0.4448 | 0.0107 | 0.0142 | 0.0035 |
| 0.2311 | 0.3217 | 0.4473 | | | | | | |
| 0.3130 | 0.3215 | 0.3655 | 0.3160 | 0.3199 | 0.3641 | 0.0029 | 0.0056 | 0.0043 |
| 0.3162 | 0.3246 | 0.3592 | | | | | | |
| 0.3188 | 0.3137 | 0.3675 | | | | | | |
| 0.4005 | 0.3004 | 0.2991 | 0.4013 | 0.2989 | 0.2998 | 0.0007 | 0.0016 | 0.0011 |
| 0.4017 | 0.2989 | 0.2994 | | | | | | |
| 0.4018 | 0.2972 | 0.3010 | | | | | | |
| 0.4716 | 0.2645 | 0.2639 | 0.4729 | 0.2746 | 0.2525 | 0.0011 | 0.0088 | 0.0099 |
| 0.4734 | 0.2791 | 0.2475 | | | | | | |
| 0.4736 | 0.2803 | 0.2461 | | | | | | |

Table S5: STDEV on measured solubilities of Benzene (1) / Cyclohexane (2) mixture with BTBACl:Cre (1:2) (3) for mole fractions x: Top layer

| Mole fraction | | | Average mole fraction | | | STDEV mole fraction | | |
|---|---|---|---|---|---|---|---|---|
| $x_1$ | $x_2$ | $x_3$ | $x_1$ | $x_2$ | $x_3$ | $x_1$ | $x_2$ | $x_3$ |
| 0.0791 | 0.9209 | 0 | 0.0789 | 0.9211 | 0 | 0.0004 | 0.0004 | 0 |
| 0.0792 | 0.9208 | 0 | | | | | | |
| 0.0785 | 0.9215 | 0 | | | | | | |
| 0.1529 | 0.8471 | 0 | 0.1527 | 0.8473 | 0 | 0.0004 | 0.0004 | 0 |
| 0.1524 | 0.8476 | 0 | | | | | | |
| 0.2312 | 0.7688 | 0 | 0.2304 | 0.7696 | 0 | 0.0007 | 0.0007 | 0 |
| 0.2299 | 0.7701 | 0 | | | | | | |
| 0.2301 | 0.7699 | 0 | | | | | | |
| 0.3240 | 0.6760 | 0 | 0.3229 | 0.6771 | 0 | 0.0011 | 0.0011 | 0 |
| 0.3219 | 0.6781 | 0 | | | | | | |
| 0.3227 | 0.6773 | 0 | | | | | | |

Table S6: STDEV on measured solubilities of Benzene (1) / Cyclohexane (2) mixture with BTBACl:Cre (1:2) (3) for mole fractions x: Bottom layer

| Mole fraction | | | Average mole fraction | | | STDEV mole fraction | | |
|---|---|---|---|---|---|---|---|---|
| $x_1$ | $x_2$ | $x_3$ | $x_1$ | $x_2$ | $x_3$ | $x_1$ | $x_2$ | $x_3$ |
| 0.1217 | 0.4815 | 0.3968 | 0.1220 | 0.4803 | 0.3977 | 0.0008 | 0.0016 | 0.0009 |
| 0.1215 | 0.4809 | 0.3976 | | | | | | |
| 0.1230 | 0.4784 | 0.3986 | | | | | | |
| 0.2336 | 0.4605 | 0.3059 | 0.2354 | 0.4531 | 0.3115 | 0.0026 | 0.0105 | 0.0079 |
| 0.2372 | 0.4457 | 0.3171 | | | | | | |
| 0.3043 | 0.4641 | 0.2316 | 0.3125 | 0.4324 | 0.2551 | 0.0071 | 0.0275 | 0.0204 |
| 0.3166 | 0.4167 | 0.2667 | | | | | | |
| 0.3166 | 0.4163 | 0.2671 | | | | | | |
| 0.3832 | 0.4081 | 0.2086 | 0.3831 | 0.4111 | 0.2059 | 0.0012 | 0.0050 | 0.0040 |
| 0.3841 | 0.4082 | 0.2076 | | | | | | |
| 0.3819 | 0.4168 | 0.2013 | | | | | | |

Table S7: STDEV on measured solubilities of Benzene (1) / Cyclohexane (2) mixture with TBPB:PTSA (1:1) (3) for mole fractions x: Top layer

| **Mole fraction** | | | **Average mole fraction** | | | **STDEV mole fraction** | | |
|---|---|---|---|---|---|---|---|---|
| $x_1$ | $x_2$ | $x_3$ | $x_1$ | $x_2$ | $x_3$ | $x_1$ | $x_2$ | $x_3$ |
| 0.0900 | 0.9100 | 0 | 0.0893 | 0.9107 | 0 | 0.0011 | 0.0011 | 0 |
| 0.0900 | 0.9100 | 0 | | | | | | |
| 0.0881 | 0.9119 | 0 | | | | | | |
| 0.1786 | 0.8214 | 0 | 0.1785 | 0.8215 | 0 | 0.0019 | 0.0019 | 0 |
| 0.1804 | 0.8196 | 0 | | | | | | |
| 0.1766 | 0.8234 | 0 | | | | | | |
| 0.2645 | 0.7355 | 0 | 0.2672 | 0.7328 | 0 | 0.0042 | 0.0042 | 0 |
| 0.2649 | 0.7351 | 0 | | | | | | |
| 0.2721 | 0.7279 | 0 | | | | | | |
| 0.3557 | 0.6443 | 0 | 0.3593 | 0.6407 | 0 | 0.0038 | 0.0038 | 0 |
| 0.3590 | 0.6410 | 0 | | | | | | |
| 0.3632 | 0.6368 | 0 | | | | | | |
| 0.4548 | 0.5452 | 0 | 0.4607 | 0.5393 | 0 | 0.0092 | 0.0092 | 0 |
| 0.4559 | 0.5441 | 0 | | | | | | |
| 0.4713 | 0.5287 | 0 | | | | | | |
| 0.5505 | 0.4495 | 0 | 0.5526 | 0.4474 | 0 | 0.0026 | 0.0026 | 0 |
| 0.5517 | 0.4483 | 0 | | | | | | |
| 0.5555 | 0.4445 | 0 | | | | | | |

Table S8: STDEV on measured solubilities of Benzene (1) / Cyclohexane (2) mixture with TBPB:PTSA (1:1) (3) for mole fractions x: Bottom layer

| Mole fraction | | | Average mole fraction | | | STDEV mole fraction | | |
|---|---|---|---|---|---|---|---|---|
| $x_1$ | $x_2$ | $x_3$ | $x_1$ | $x_2$ | $x_3$ | $x_1$ | $x_2$ | $x_3$ |
| 0.1038 | 0.2134 | 0.6827 | 0.1064 | 0.2041 | 0.6895 | 0.0040 | 0.0123 | 0.0139 |
| 0.1043 | 0.1901 | 0.7056 | | | | | | |
| 0.1111 | 0.2086 | 0.6803 | | | | | | |
| 0.2016 | 0.1921 | 0.6063 | 0.2018 | 0.1899 | 0.6083 | 0.0064 | 0.0048 | 0.0109 |
| 0.1955 | 0.1845 | 0.6201 | | | | | | |
| 0.2082 | 0.1933 | 0.5986 | | | | | | |
| 0.2989 | 0.2113 | 0.4898 | 0.2925 | 0.1955 | 0.5120 | 0.0111 | 0.0164 | 0.0267 |
| 0.2989 | 0.1965 | 0.5046 | | | | | | |
| 0.2797 | 0.1786 | 0.5417 | | | | | | |
| 0.3760 | 0.1817 | 0.4423 | 0.3677 | 0.1752 | 0.4571 | 0.0089 | 0.0063 | 0.0152 |
| 0.3687 | 0.1748 | 0.4565 | | | | | | |
| 0.3583 | 0.1691 | 0.4726 | | | | | | |
| 0.4263 | 0.1641 | 0.4096 | 0.4093 | 0.1565 | 0.4342 | 0.0271 | 0.0103 | 0.0373 |
| 0.4235 | 0.1607 | 0.4158 | | | | | | |
| 0.3781 | 0.1448 | 0.4771 | | | | | | |
| 0.5158 | 0.1639 | 0.3202 | 0.5116 | 0.1589 | 0.3295 | 0.0050 | 0.0044 | 0.0089 |
| 0.5129 | 0.1570 | 0.3301 | | | | | | |
| 0.5062 | 0.1558 | 0.3380 | | | | | | |

Table S9: Molar ternary compositions, benzene distribution ratio ($D_{Bz}$) and solvent selectivity (S) from previous works involving organic solvents, ILs and DESs. $x_1$, $x_2$ and $x_3$ represents the molar composition of benzene, cyclohexane and solvents, respectively.

| **Top layer** | | | **Bottom layer** | | | $D_{Bz}$ | S |
|---|---|---|---|---|---|---|---|
| $x_1$ | $x_2$ | $x_3$ | $x_1$ | $x_2$ | $x_3$ | | |
| Benzene (1) + cyclohexane + sulfolane (3) | | | | | | | |
| 0.036 | 0.963 | 1x10$^{-3}$ | 0.015 | 0.027 | 0.958 | 0.417 | 14.86 |
| 0.133 | 0.856 | 0.011 | 0.069 | 0.037 | 0.894 | 0.519 | 12.00 |
| 0.158 | 0.829 | 0.013 | 0.086 | 0.038 | 0.876 | 0.544 | 11.87 |
| 0.217 | 0.764 | 0.019 | 0.118 | 0.039 | 0.843 | 0.544 | 10.65 |
| 0.268 | 0.701 | 0.031 | 0.156 | 0.05 | 0.794 | 0.582 | 8.16 |
| 0.31 | 0.649 | 0.041 | 0.186 | 0.055 | 0.759 | 0.6 | 7.08 |
| 0.356 | 0.586 | 0.058 | 0.225 | 0.065 | 0.71 | 0.632 | 5.698 |
| 0.394 | 0.53 | 0.076 | 0.263 | 0.078 | 0.659 | 0.667 | 4.54 |
| 0.433 | 0.458 | 0.109 | 0.314 | 0.099 | 0.587 | 0.725 | 3.35 |
| 0.457 | 0.387 | 0.156 | 0.361 | 0.13 | 0.509 | 0.79 | 2.35 |
| Benzene (1) + cyclohexane + ethylene glycol (3) | | | | | | | |
| 0.258 | 0.74 | 0.002 | 0.0348 | 0.0021 | 0.965 | 0.13 | 47.53 |
| 0.464 | 0.53 | 0.004 | 0.086 | 0.0021 | 0.912 | 0.19 | 46.78 |
| 0.66 | 0.33 | 0.005 | 0.172 | 0.0045 | 0.823 | 0.26 | 19.11 |
| 0.831 | 0.161 | 0.008 | 0.221 | 0.0045 | 0.774 | 0.26 | 9.51 |
| Benzene (1) + cyclohexane + dimethylformamide (3) | | | | | | | |
| 0.0243 | 0.8907 | 0.085 | 0.0219 | 0.2677 | 0.7105 | 0.90123 | 2.99862 |
| 0.0758 | 0.8112 | 0.113 | 0.0652 | 0.3321 | 0.6027 | 0.86016 | 2.10106 |
| 0.1106 | 0.6542 | 0.2352 | 0.1018 | 0.3752 | 0.523 | 0.92043 | 1.60487 |
| Benzene (1) + cyclohexane (2) + TBABr:Sulf (1:7) (3) | | | | | | | |
| 0.101 | 0.899 | 0.000 | 0.020 | 0.013 | 0.967 | 0.196 | 13.354 |
| 0.194 | 0.806 | 0.000 | 0.041 | 0.014 | 0.944 | 0.212 | 11.966 |
| 0.284 | 0.716 | 0.000 | 0.059 | 0.015 | 0.926 | 0.209 | 9.859 |
| 0.366 | 0.634 | 0.000 | 0.081 | 0.017 | 0.902 | 0.222 | 8.164 |
| 0.471 | 0.529 | 0.000 | 0.104 | 0.018 | 0.878 | 0.222 | 6.500 |
| 0.562 | 0.438 | 0.000 | 0.134 | 0.021 | 0.845 | 0.238 | 4.902 |
| Benzene (1) + cyclohexane (2) + TBABr:TEG (1:4) (3) | | | | | | | |
| 0.099 | 0.901 | 0.000 | 0.017 | 0.018 | 0.965 | 0.171 | 8.322 |
| 0.203 | 0.797 | 0.000 | 0.028 | 0.016 | 0.956 | 0.136 | 6.582 |
| 0.279 | 0.721 | 0.000 | 0.036 | 0.019 | 0.944 | 0.130 | 4.831 |
| 0.367 | 0.633 | 0.000 | 0.055 | 0.013 | 0.931 | 0.150 | 7.039 |
| 0.478 | 0.522 | 0.000 | 0.070 | 0.016 | 0.914 | 0.145 | 4.702 |
| 0.576 | 0.424 | 0.000 | 0.086 | 0.015 | 0.899 | 0.149 | 4.257 |
| 0.678 | 0.322 | 0.000 | 0.104 | 0.016 | 0.880 | 0.153 | 3.073 |
| 0.774 | 0.226 | 0.000 | 0.128 | 0.016 | 0.856 | 0.166 | 2.301 |
| Benzene (1) + cyclohexane (2) + MTPPBr:TEG (1:4) (3) | | | | | | | |
| 0.106 | 0.894 | 0.000 | 0.011 | 0.010 | 0.979 | 0.105 | 12.882 |
| 0.197 | 0.803 | 0.000 | 0.023 | 0.007 | 0.971 | 0.115 | 14.102 |
| 0.294 | 0.706 | 0.000 | 0.033 | 0.009 | 0.958 | 0.113 | 9.220 |
| 0.387 | 0.613 | 0.000 | 0.041 | 0.005 | 0.954 | 0.106 | 12.846 |
| 0.492 | 0.508 | 0.000 | 0.054 | 0.007 | 0.939 | 0.109 | 7.868 |
| 0.590 | 0.410 | 0.000 | 0.066 | 0.006 | 0.928 | 0.112 | 7.630 |

| | | | | | | | |
|---|---|---|---|---|---|---|---|
| 0.692 | 0.308 | 0.000 | 0.081 | 0.006 | 0.914 | 0.117 | 6.190 |
| 0.828 | 0.172 | 0.000 | 0.103 | 0.005 | 0.893 | 0.124 | 4.493 |
| Benzene (1) + cyclohexane (2) + MTPPBr:PD (1:4) (3) | | | | | | | |
| 0.110 | 0.890 | 0.000 | 0.010 | 0.010 | 0.980 | 0.093 | 8.024 |
| 0.214 | 0.786 | 0.000 | 0.018 | 0.010 | 0.972 | 0.083 | 6.578 |
| 0.298 | 0.702 | 0.000 | 0.023 | 0.010 | 0.967 | 0.077 | 5.526 |
| 0.403 | 0.597 | 0.000 | 0.033 | 0.009 | 0.958 | 0.081 | 5.217 |
| 0.502 | 0.498 | 0.000 | 0.044 | 0.009 | 0.947 | 0.087 | 4.630 |
| 0.602 | 0.398 | 0.000 | 0.055 | 0.008 | 0.938 | 0.091 | 4.741 |
| 0.703 | 0.297 | 0.000 | 0.061 | 0.008 | 0.931 | 0.087 | 3.208 |
| 0.792 | 0.208 | 0.000 | 0.077 | 0.009 | 0.914 | 0.097 | 2.186 |
| Benzene (1) + cyclohexane (2) + ChCl:TEG (1:4) (3) | | | | | | | |
| 0.094 | 0.906 | 0.000 | 0.007 | 0.007 | 0.986 | 0.074 | 9.130 |
| 0.197 | 0.803 | 0.000 | 0.014 | 0.006 | 0.980 | 0.070 | 9.613 |
| 0.292 | 0.708 | 0.000 | 0.020 | 0.004 | 0.976 | 0.069 | 12.091 |
| 0.399 | 0.601 | 0.000 | 0.027 | 0.004 | 0.969 | 0.067 | 9.032 |
| 0.502 | 0.498 | 0.000 | 0.032 | 0.004 | 0.964 | 0.064 | 8.228 |
| 0.607 | 0.393 | 0.000 | 0.040 | 0.004 | 0.955 | 0.067 | 6.100 |
| 0.704 | 0.296 | 0.000 | 0.048 | 0.004 | 0.948 | 0.068 | 4.896 |
| 0.809 | 0.191 | 0.000 | 0.057 | 0.003 | 0.940 | 0.071 | 4.749 |
| Benzene (1) + cyclohexane (2) + [C2mim][Ac] (3) | | | | | | | |
| 0.085 | 0.915 | 0.000 | 0.036 | 0.021 | 0.943 | 0.42 | 18.34 |
| 0.175 | 0.825 | 0.000 | 0.085 | 0.069 | 0.847 | 0.48 | 10.33 |
| 0.264 | 0.736 | 0.000 | 0.140 | 0.040 | 0.820 | 0.53 | 10.15 |
| 0.360 | 0.640 | 0.000 | 0.180 | 0.034 | 0.776 | 0.50 | 9.56 |
| 0.462 | 0.538 | 0.000 | 0.212 | 0.033 | 0.755 | 0.46 | 7.44 |
| 0.561 | 0.439 | 0.000 | 0.218 | 0.040 | 0.742 | 0.39 | 4.26 |
| Benzene (1) + cyclohexane (2) + [C2mim][SCN] (3) | | | | | | | |
| 0.082 | 0.918 | 0.000 | 0.038 | 0.010 | 0.953 | 0.46 | 44.71 |
| 0.168 | 0.832 | 0.000 | 0.077 | 0.012 | 0.910 | 0.46 | 31.18 |
| 0.259 | 0.741 | 0.000 | 0.114 | 0.010 | 0.876 | 0.44 | 32.73 |
| 0.351 | 0.649 | 0.000 | 0.149 | 0.009 | 0.842 | 0.42 | 30.94 |
| 0.450 | 0.550 | 0.000 | 0.184 | 0.008 | 0.808 | 0.41 | 26.53 |
| 0.553 | 0.447 | 0.000 | 0.222 | 0.007 | 0.771 | 0.40 | 25.22 |
| Benzene (1) + cyclohexane (2) + [C2mim][N(CN)2] (3) | | | | | | | |
| 0.073 | 0.927 | 0.000 | 0.049 | 0.020 | 0.932 | 0.67 | 31.85 |
| 0.158 | 0.842 | 0.000 | 0.095 | 0.018 | 0.886 | 0.61 | 27.94 |
| 0.247 | 0.753 | 0.000 | 0.141 | 0.016 | 0.844 | 0.57 | 27.48 |
| 0.339 | 0.661 | 0.000 | 0.184 | 0.015 | 0.801 | 0.54 | 23.51 |
| 0.426 | 0.574 | 0.000 | 0.232 | 0.016 | 0.752 | 0.54 | 19.23 |
| 0.522 | 0.478 | 0.000 | 0.275 | 0.015 | 0.710 | 0.53 | 16.84 |
| Benzene (1) + cyclohexane (2) + [C2mim][Tf2N] (3) | | | | | | | |
| 0.078 | 0.922 | 0.000 | 0.095 | 0.059 | 0.846 | 1.22 | 19.09 |
| 0.157 | 0.843 | 0.000 | 0.185 | 0.058 | 0.757 | 1.18 | 17.14 |
| 0.243 | 0.757 | 0.000 | 0.259 | 0.057 | 0.685 | 1.07 | 14.28 |
| 0.332 | 0.668 | 0.000 | 0.331 | 0.054 | 0.615 | 1.00 | 12.36 |
| 0.417 | 0.583 | 0.000 | 0.396 | 0.054 | 0.549 | 0.95 | 10.24 |
| 0.518 | 0.482 | 0.000 | 0.459 | 0.054 | 0.487 | 0.89 | 7.91 |

| | | | | | | | |
|---|---|---|---|---|---|---|---|
| Benzene (1) + cyclohexane (2) + [C4mim][Tf2N] (3) | | | | | | | |
| 0.04 | 0.96 | 0.000 | 0.066 | 0.162 | 0.772 | 0.168 | 9.78 |
| 0.124 | 0.876 | 0.000 | 0.186 | 0.156 | 0.658 | 0.178 | 8.42 |
| 0.177 | 0.823 | 0.000 | 0.258 | 0.153 | 0.589 | 0.18 | 7.84 |
| 0.23 | 0.77 | 0.000 | 0.301 | 0.146 | 0.553 | 0.19 | 6.90 |
| 0.277 | 0.723 | 0.000 | 0.355 | 0.139 | 0.506 | 0.19 | 6.67 |
| 0.352 | 0.648 | 0.000 | 0.413 | 0.13 | 0.457 | 0.20 | 5.85 |
| 0.439 | 0.561 | 0.000 | 0.478 | 0.117 | 0.405 | 0.21 | 5.22 |
| 0.524 | 0.476 | 0.000 | 0.537 | 0.104 | 0.359 | 0.22 | 4.69 |
| 0.62 | 0.38 | 0.000 | 0.595 | 0.09 | 0.315 | 0.24 | 4.05 |
| 0.784 | 0.216 | 0.000 | 0.705 | 0.051 | 0.244 | 0.24 | 3.81 |
| Benzene (1) + cyclohexane (2) + [C4mim][PF6] (3) | | | | | | | |
| 0.0392 | 0.9608 | 0.000 | 0.0542 | 0.0269 | 0.9189 | 1.384 | 49.4 |
| 0.0795 | 0.9205 | 0.000 | 0.1098 | 0.0319 | 0.8583 | 1.382 | 39.9 |
| 0.1628 | 0.8372 | 0.000 | 0.1647 | 0.0368 | 0.7985 | 1.012 | 23 |
| 0.2481 | 0.7519 | 0.000 | 0.2375 | 0.0327 | 0.7298 | 0.957 | 22 |
| 0.3507 | 0.6493 | 0.000 | 0.2723 | 0.0395 | 0.6882 | 0.776 | 12.8 |
| 0.449 | 0.551 | 0.000 | 0.324 | 0.0311 | 0.6449 | 0.721 | 12.8 |
| 0.6462 | 0.3538 | 0.000 | 0.4321 | 0.0245 | 0.5434 | 0.669 | 9.7 |
| 0.8724 | 0.1276 | 0.000 | 0.54 | 0.012 | 0.448 | 0.619 | 6.6 |
| Benzene (1) + cyclohexane (2) + [C4mim][BF4] (3) | | | | | | | |
| 0.046 | 0.954 | 0.000 | 0.031 | 0.008 | 0.961 | 0.67 | 80.36 |
| 0.092 | 0.908 | 0.000 | 0.066 | 0.012 | 0.922 | 0.71 | 54.28 |
| 0.165 | 0.835 | 0.000 | 0.102 | 0.019 | 0.879 | 0.62 | 27.17 |
| 0.245 | 0.755 | 0.000 | 0.153 | 0.026 | 0.821 | 0.62 | 18.13 |
| 0.399 | 0.601 | 0.000 | 0.245 | 0.034 | 0.721 | 0.61 | 10.85 |
| 0.523 | 0.477 | 0.000 | 0.326 | 0.042 | 0.632 | 0.62 | 7.08 |
| 0.682 | 0.318 | 0.000 | 0.465 | 0.057 | 0.478 | 0.68 | 3.80 |
| 0.837 | 0.163 | 0.000 | 0.646 | 0.039 | 0.315 | 0.77 | 3.22 |
| Benzene (1) + cyclohexane (2) + [C2mim][EtSO4] (3) | | | | | | | |
| 0.054 | 0.946 | 0.000 | 0.034 | 0.03 | 0.936 | 0.63 | 19.85 |
| 0.11 | 0.89 | 0.000 | 0.061 | 0.031 | 0.908 | 0.55 | 15.92 |
| 0.166 | 0.834 | 0.000 | 0.084 | 0.031 | 0.885 | 0.51 | 13.61 |
| 0.223 | 0.777 | 0.000 | 0.112 | 0.03 | 0.858 | 0.50 | 13.00 |
| 0.349 | 0.651 | 0.000 | 0.171 | 0.026 | 0.803 | 0.49 | 12.27 |
| 0.47 | 0.53 | 0.000 | 0.222 | 0.021 | 0.757 | 0.47 | 11.92 |
| 0.591 | 0.409 | 0.000 | 0.273 | 0.017 | 0.71 | 0.46 | 11.11 |
| 0.726 | 0.274 | 0.000 | 0.33 | 0.011 | 0.659 | 0.45 | 11.32 |
| 0.862 | 0.138 | 0.000 | 0.389 | 0.006 | 0.605 | 0.45 | 10.38 |
| Benzene (1) + cyclohexane (2) + [C4mim][AlCl4] (3) | | | | | | | |
| 0.0438 | 0.9079 | 0.0483 | 0.055 | 0.0524 | 0.8926 | 1.26 | 21.76 |
| 0.0887 | 0.8616 | 0.0497 | 0.1099 | 0.0498 | 0.8403 | 1.24 | 21.44 |
| 0.1444 | 0.807 | 0.0486 | 0.1536 | 0.0419 | 0.8045 | 1.06 | 20.45 |
| 0.1948 | 0.7533 | 0.0519 | 0.2033 | 0.0457 | 0.751 | 1.04 | 17.20 |
| 0.2525 | 0.6857 | 0.0618 | 0.2471 | 0.0415 | 0.7114 | 0.97 | 16.17 |
| 0.3035 | 0.6214 | 0.0751 | 0.2972 | 0.0366 | 0.6662 | 0.98 | 16.63 |
| 0.4122 | 0.5008 | 0.087 | 0.3759 | 0.0335 | 0.5906 | 0.91 | 13.63 |

## References


[1] F. S. Oliveira, A. B. Pereiro, L. P. Rebelo, and I. M. Marrucho, "Deep eutectic solvents as extraction media for azeotropic mixtures," *Green Chemistry,* vol. 15, no. 5, pp. 1326-1330, 2013.

[2] D. J. van Osch, L. F. Zubeir, A. van den Bruinhorst, M. A. Rocha, and M. C. Kroon, "Hydrophobic deep eutectic solvents as water-immiscible extractants," *Green Chemistry,* vol. 17, no. 9, pp. 4518-4521, 2015.

[3] T. E. Phelps, N. Bhawawet, S. S. Jurisson, and G. A. Baker, "Efficient and selective extraction of 99mTcO4–from aqueous media using hydrophobic deep eutectic solvents," *ACS Sustainable Chemistry & Engineering,* vol. 6, no. 11, pp. 13656-13661, 2018.

[4] N. Schaeffer, M. A. Martins, C. M. Neves, S. P. Pinho, and J. A. Coutinho, "Sustainable hydrophobic terpene-based eutectic solvents for the extraction and separation of metals," *Chemical Communications,* vol. 54, no. 58, pp. 8104-8107, 2018.

[5] E. Tereshatov, M. Y. Boltoeva, and C. Folden, "First evidence of metal transfer into hydrophobic deep eutectic and low-transition-temperature mixtures: indium extraction from hydrochloric and oxalic acids," *Green Chem.,* vol. 18, no. 17, pp. 4616-4622, 2016.

[6] G. Almustafa, R. Sulaiman, M. Kumar, I. Adeyemi, H. A. Arafat, and I. AlNashef, "Boron extraction from aqueous medium using novel hydrophobic deep eutectic solvents," *Chemical Engineering Journal,* vol. 395, p. 125173, 2020.

[7] G. Zante, A. Braun, A. Masmoudi, R. Barillon, D. Trebouet, and M. Boltoeva, "Solvent extraction fractionation of manganese, cobalt, nickel and lithium using ionic liquids and deep eutectic solvents," *Miner. Eng.,* vol. 156, p. 106512, 2020.

[8] X. Liu, B. Gao, Y. Jiang, N. Ai, and D. Deng, "Solubilities and thermodynamic properties of carbon dioxide in guaiacol-based deep eutectic solvents," *Journal of Chemical & Engineering Data,* vol. 62, no. 4, pp. 1448-1455, 2017.

[9] T. Altamash *et al.*, "Gas solubility and rheological behavior study of betaine and alanine based natural deep eutectic solvents (NADES)," *J. Mol. Liq.,* vol. 256, pp. 286-295, 2018.

[10] S. Sarmad, Y. Xie, J.-P. Mikkola, and X. Ji, "Screening of deep eutectic solvents (DESs) as green CO 2 sorbents: from solubility to viscosity," *New J. Chem.,* vol. 41, no. 1, pp. 290-301, 2017.

[11] X. Li, M. Hou, B. Han, X. Wang, and L. Zou, "Solubility of CO2 in a choline chloride+ urea eutectic mixture," *J. Chem. Eng. Data,* vol. 53, no. 2, pp. 548-550, 2008.

[12] R. B. Leron and M.-H. Li, "Solubility of carbon dioxide in a choline chloride–ethylene glycol based deep eutectic solvent," *Thermochimica acta,* vol. 551, pp. 14-19, 2013.

[13] G. R. Jenkin *et al.*, "The application of deep eutectic solvent ionic liquids for environmentally-friendly dissolution and recovery of precious metals," *Miner. Eng.,* vol. 87, pp. 18-24, 2016.

[14] A. P. Abbott, G. Capper, K. J. McKenzie, and K. S. Ryder, "Electrodeposition of zinc–tin alloys from deep eutectic solvents based on choline chloride," *Journal of Electroanalytical Chemistry,* vol. 599, no. 2, pp. 288-294, 2007.

[15] Y. You, C. Gu, X. Wang, and J. Tu, "Electrodeposition of Ni–Co alloys from a deep eutectic solvent," *Surface and Coatings Technology,* vol. 206, no. 17, pp. 3632-3638, 2012.

[16] J. C. Malaquias, M. Steichen, M. Thomassey, and P. J. Dale, "Electrodeposition of Cu–In alloys from a choline chloride based deep eutectic solvent for photovoltaic applications," *Electrochim. Acta,* vol. 103, pp. 15-22, 2013.

[17] E. Gómez, P. Cojocaru, L. Magagnin, and E. Valles, "Electrodeposition of Co, Sm and SmCo from a deep eutectic solvent," *Journal of electroanalytical chemistry,* vol. 658, no. 1-2, pp. 18-24, 2011.

[18] C. Gu and J. Tu, "One-step fabrication of nanostructured Ni film with lotus effect from deep eutectic solvent," *Langmuir,* vol. 27, no. 16, pp. 10132-10140, 2011.

[19] A. H. Whitehead, M. Pölzler, and B. Gollas, "Zinc electrodeposition from a deep eutectic system containing choline chloride and ethylene glycol," *Journal of the Electrochemical Society,* vol. 157, no. 6, p. D328, 2010.

[20] A. P. Abbott, G. Capper, D. L. Davies, K. J. McKenzie, and S. U. Obi, "Solubility of metal oxides in deep eutectic solvents based on choline chloride," *J. Chem. Eng. Data,* vol. 51, no. 4, pp. 1280-1282, 2006.

[21] V. S. Raghuwanshi, M. Ochmann, A. Hoell, F. Polzer, and K. Rademann, "Deep eutectic solvents for the self-assembly of gold nanoparticles: a SAXS, UV–Vis, and TEM investigation," *Langmuir,* vol. 30, no. 21, pp. 6038-6046, 2014.

[22] Y. Huang *et al.*, "Synthesis and characterization of CuCl nanoparticles in deep eutectic solvents," *Particulate Science and Technology,* vol. 31, no. 1, pp. 81-84, 2013.

[23] L. Adhikari, N. E. Larm, and G. A. Baker, "Argentous deep eutectic solvent approach for scaling up the production of colloidal silver nanocrystals," *ACS Sustainable Chem. Eng.,* vol. 7, no. 13, pp. 11036-11043, 2019.

[24] M. Hayyan, F. S. Mjalli, M. A. Hashim, and I. M. AlNashef, "A novel technique for separating glycerine from palm oil-based biodiesel using ionic liquids," *Fuel Processing Technology,* vol. 91, no. 1, pp. 116-120, 2010.

[25] A. P. Abbott, P. M. Cullis, M. J. Gibson, R. C. Harris, and E. Raven, "Extraction of glycerol from biodiesel into a eutectic based ionic liquid," *Green Chem.,* vol. 9, no. 8, pp. 868-872, 2007.

[26] H. Zhao, G. A. Baker, and S. Holmes, "New eutectic ionic liquids for lipase activation and enzymatic preparation of biodiesel," *Organic & biomolecular chemistry,* vol. 9, no. 6, pp. 1908-1916, 2011.

[27] H. Zhao, C. Zhang, and T. D. Crittle, "Choline-based deep eutectic solvents for enzymatic preparation of biodiesel from soybean oil," *Journal of Molecular Catalysis B: Enzymatic,* vol. 85, pp. 243-247, 2013.

[28] Q. Xu *et al.*, "A deep eutectic solvent (DES) electrolyte-based vanadium-iron redox flow battery enabling higher specific capacity and improved thermal stability," *Electrochim. Acta,* vol. 293, pp. 426-431, 2019.

[29] H.-R. Jhong, D. S.-H. Wong, C.-C. Wan, Y.-Y. Wang, and T.-C. Wei, "A novel deep eutectic solvent-based ionic liquid used as electrolyte for dye-sensitized solar cells," *Electrochemistry Communications,* vol. 11, no. 1, pp. 209-211, 2009.

[30] C. L. Boldrini, N. Manfredi, F. M. Perna, V. Trifiletti, V. Capriati, and A. Abbotto, "Dye-sensitized solar cells that use an aqueous choline chloride-based deep eutectic solvent as effective electrolyte solution," *Energy Technology,* vol. 5, no. 2, pp. 345-353, 2017.

[31] B. Bozzini, B. Busson, C. Humbert, C. Mele, and A. Tadjeddine, "Electrochemical fabrication of nanoporous gold decorated with manganese oxide nanowires from eutectic urea/choline chloride ionic liquid. Part III− Electrodeposition of Au–Mn: a study based on in situ Sum-Frequency Generation and Raman spectroscopies," *Electrochim. Acta,* vol. 218, pp. 208-215, 2016.

[32] Y.-S. Lu, W.-Y. Pan, T.-C. Hung, and Y.-T. Hsieh, "Electrodeposition of silver in a ternary deep eutectic solvent and the electrochemical sensing ability of the Ag-modified electrode for nitrofurazone," *Langmuir,* vol. 36, no. 38, pp. 11358-11365, 2020.

[33] F. Milano *et al.*, "Functional enzymes in nonaqueous environment: the case of photosynthetic reaction centers in deep eutectic solvents," *ACS Sustainable Chemistry & Engineering,* vol. 5, no. 9, pp. 7768-7776, 2017.
